# Distinct strategies for intravascular triglyceride metabolism in hearts of mammals and lower vertebrate species

**DOI:** 10.1172/jci.insight.184940

**Published:** 2024-09-17

**Authors:** Le Phuong Nguyen, Wenxin Song, Ye Yang, Anh P. Tran, Thomas A. Weston, Hyesoo Jung, Yiping Tu, Paul H. Kim, Joonyoung R. Kim, Katherine Xie, Rachel G. Yu, Julia Scheithauer, Ashley M. Presnell, Michael Ploug, Gabriel Birrane, Hannah Arnold, Katarzyna Koltowska, Maarja A. Mäe, Christer Betsholtz, Liqun He, Jeffrey L. Goodwin, Anne P. Beigneux, Loren G. Fong, Stephen G. Young

**Affiliations:** 1 Department of Medicine and; 2 Human Genetics, David Geffen School of Medicine, UCLA, Los Angeles, California, USA.; 3 Finsen Laboratory, Copenhagen University Hospital-Rigshospitalet, Copenhagen, Denmark.; 4 Finsen Laboratory, Biotech Research and Innovation Centre, University of Copenhagen, Copenhagen, Denmark.; 5 Division of Experimental Medicine, Beth Israel Deaconess Medical Center, Boston, Massachusetts, USA.; 6 Department of Immunology, Genetics, and Pathology, Rudbeck Laboratory, Uppsala University, Uppsala, Sweden.; 7 Beijer Gene and Neuro Laboratory, Uppsala University, Uppsala, Sweden.; 8 Department of Medicine-Huddinge, Karolinska Institute Campus Flemingsberg, Huddinge, Sweden.; 9 Division of Laboratory Animal Medicine, David Geffen School of Medicine, UCLA, Los Angeles, California, USA.

**Keywords:** Cardiology, Metabolism, Endothelial cells, Lipoproteins

## Abstract

Lipoprotein lipase (LPL) and multiple regulators of LPL activity (e.g., APOC2 and ANGPTL4) are present in all vertebrates, but GPIHBP1—the endothelial cell (EC) protein that captures LPL within the subendothelial spaces and transports it to its site of action in the capillary lumen—is present in mammals but in not chickens or other lower vertebrates. In mammals, GPIHBP1 deficiency causes severe hypertriglyceridemia, but chickens maintain low triglyceride levels despite the absence of GPIHBP1. To understand intravascular lipolysis in lower vertebrates, we examined LPL expression in mouse and chicken hearts. In both species, LPL was abundant on capillaries, but the distribution of *Lpl* transcripts was strikingly different. In mouse hearts, *Lpl* transcripts were extremely abundant in cardiomyocytes but were barely detectable in capillary ECs. In chicken hearts, *Lpl* transcripts were absent in cardiomyocytes but abundant in capillary ECs. In zebrafish hearts, *lpl* transcripts were also in capillary ECs but not cardiomyocytes. In both mouse and chicken hearts, LPL was present, as judged by immunogold electron microscopy, in the glycocalyx of capillary ECs. Thus, mammals produce LPL in cardiomyocytes and rely on GPIHBP1 to transport the LPL into capillaries, whereas lower vertebrates produce LPL directly in capillary ECs, rendering an LPL transporter unnecessary.

## Abstract 2

Intravascular triglyceride hydrolysis is crucial for delivering lipids to the heart. We discovered different strategies for heart triglyceride metabolism in mammals and lower vertebrates.

## Introduction

Lipoprotein lipase (LPL) is essential for the intravascular lipolytic processing of triglyceride-rich (TG-rich) lipoproteins (TRLs) (1, 2). In mice, cats, and humans, LPL deficiency markedly impairs TRL processing, resulting in severe hypertriglyceridemia (chylomicronemia) (3-5). In fish and birds (nonmammalian vertebrates, frequently called lower vertebrates), LPL is also important. When chickens were given an intravenous injection of an inhibitory LPL-specific antibody, TG clearance from the plasma was nearly abolished, resulting in severe hypertriglyceridemia (6). Also, LPL deficiency in zebrafish causes severe hypertriglyceridemia, with plasma TG levels over 2,000 mg/dL (7). Not surprisingly, LPL’s primary structure is conserved in vertebrates (e.g., there is 76% amino acid similarity between mouse and chicken LPL and 63% similarity between mouse and zebrafish LPL). Other proteins that regulate LPL-mediated TRL processing (*e.g*., APOC2, APOC3, ANGPTL4, and ANGPTL3) are also conserved in mammals and lower vertebrates; however, there is a noteworthy exception. GPIHBP1, the endothelial cell (EC) protein that moves LPL to its site of action in the capillary lumen, is present in mammals (including the egg-laying platypus) but is absent in lower vertebrates (8).

In human and mouse hearts, LPL is expressed at high levels in cardiomyocytes, as judged by in situ hybridization (ISH) studies with radioactive probes (9, 10). The LPL secreted by cardiomyocytes is captured by GPIHBP1 on the abluminal plasma membrane of ECs and shuttled to the luminal plasma membrane of capillary ECs (11). In mice, GPIHBP1 is abundant on capillary ECs in the heart, as judged by confocal microscopy, and is undetectable on ECs of venules, arterioles, and larger blood vessels (11-14). LPL is located on capillaries, colocalizing with GPIHBP1. In the mouse heart, much of the LPL that GPIHBP1 moves into capillaries detaches and is captured within the HSPG-rich glycocalyx lining of capillary ECs (14). The LPL within the glycocalyx mediates TRL margination along the luminal surface of capillaries and participates in the lipolytic processing of TRLs (14).

In *Gpihbp1*^–/–^ mice, LPL is stranded within the interstitial spaces and never reaches the capillary lumen, resulting in severe hypertriglyceridemia (11, 13, 14). The plasma TG levels in chow-fed *Gpihbp1*^–/–^ mice are 2,500–3,000 mg/dL (11, 15) but can reach 30,000 mg/dL on a high-fat diet (16). *GPIHBP1* deficiency in humans causes lifelong hypertriglyceridemia, with plasma TG levels greater than 1,500 mg/dL (17–20). An infant with *GPIHBP1* deficiency had plasma TG levels greater than 25,000 mg/dL (21). Some acquired forms of hypertriglyceridemia are caused by inhibitory GPIHBP1 autoantibodies (22–24). In those patients, the plasma TG levels are typically greater than 1,500 mg/dL (22–24). The profound hypertriglyceridemia in *Gpihbp1*^–/–^ mice contrasts with the situation in chickens, where GPIHBP1 is absent but plasma TG levels are less than 100 mg/dL, even on a fat-enriched diet (25, 26). Studies of TRL processing in chickens are quite limited, but it is clear that TG processing is robust (6), that LPL expression in chicken hearts is regulated during fasting and refeeding (27), and that TRL-derived fatty acids are used as fuel in the heart (28–30).

He et al. (8) reported that LPL was detectable by IHC on chicken heart capillaries. They also found that an injection of heparin into an isolated perfused chicken heart releases catalytically active LPL into the perfusate, suggesting that the LPL in the chicken heart is located inside blood vessels. These findings prompted speculation that chickens might have a yet-to-be-discovered, transporter (distinct from GPIHBP1) that moves cardiomyocyte-derived LPL into blood vessels (8). That speculation, however, was not particularly satisfying because it seemed rather unlikely that mammalian evolution would have created an entirely new transporter (namely GPIHBP1) to shuttle a conserved LPL molecule across capillary ECs. In the current studies, we sought further insights into LPL biology in chickens. We wanted to confirm, with a newly developed antibody against chicken LPL, the existence of LPL within chicken heart capillaries; we also wanted to define the location of *Lpl* transcripts in mouse and chicken hearts. Additionally, we wanted to determine whether the LPL in the chicken heart is confined to capillaries and absent from larger blood vessels, as it is in the mouse heart (11–14). Finally, we were interested in determining whether the LPL in the chicken heart reaches the glycocalyx of capillary ECs, as is the case in the mouse heart (14).

## Results

**Figure 1:**
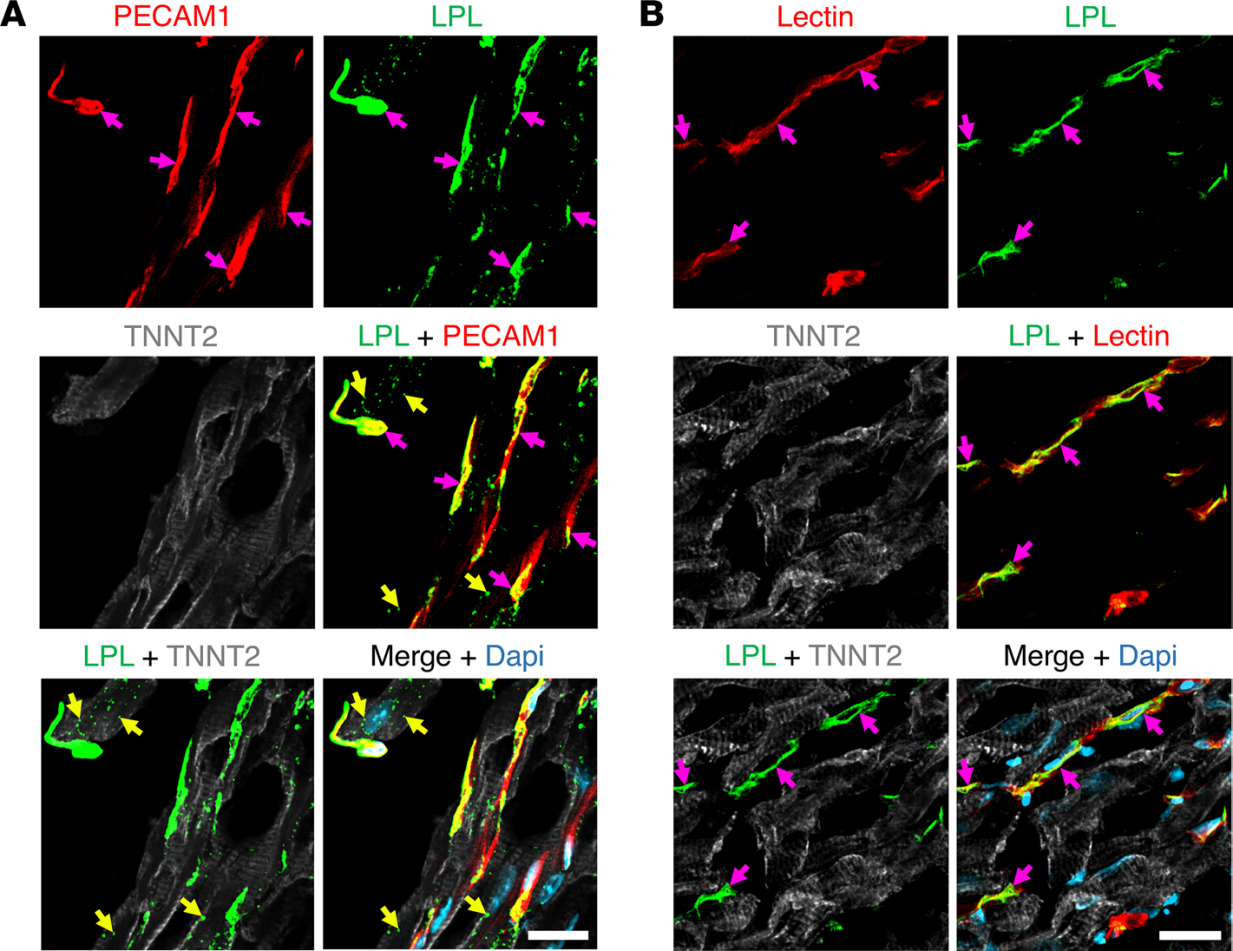
IHC studies on the localization of LPL in mouse and chicken hearts. Mouse and chicken heart cryosections were stained with antibodies against cardiac troponin T (TNNT2, white) and either a mouse LPL–specific rabbit antibody (3174) or a chicken LPL–specific rabbit antibody (4727). Nuclei were stained with Dapi (blue). (**A**) A heart section from a mouse that had been injected intravenously with an Alexa Fluor 488–labeled PECAM1-specific monoclonal antibody (2H8, red). Mouse LPL (green) was detected on PECAM1-positive capillaries (*pink* arrows) and inside cardiomyocytes (yellow arrows). (**B**) Heart section from a chicken that had been injected intravenously with a fluorescein-labeled lectin (Lens culinaris agglutinin, red). Chicken LPL (green) was detected on capillary ECs (pink arrows); amounts of LPL inside chicken cardiomyocytes were negligible or absent. Shown here are representative images from independent experiments with 4 mice and 4 chickens. Scale bar: 20 μm.

We expressed chicken LPL (cLPL) in insect cells (31) and then used the recombinant protein to produce a rabbit polyclonal antibody against cLPL (Supplemental Figure 1; supplemental material available online with this article; https://doi.org/10.1172/jci.insight.184940DS1). The cLPL-specific antibody and a rabbit polyclonal antibody against mouse LPL (mLPL) (13, 14) were used to compare the location of LPL in mouse and chicken hearts. In our studies of the mouse heart, we first marked the luminal surface of blood vessels by giving mice an intravenous injection of an Alexa Fluor–labeled monoclonal antibody (mAb) against PECAM1; we then stained cryosections of mouse heart with the mLPL-specific antibody. Confocal microscopy revealed that mLPL was located on PECAM1-positive capillaries. mLPL was also detectable in cardiomyocytes (identified with an antibody against cardiac troponin T [TNNT2]) (Figure 1A and Supplemental Figure 2A). For our studies of the chicken heart, we marked the luminal surface of blood vessels with an intravenous injection of a fluorescein-labeled lectin (*Lens culinaris agglutinin*) and then stained sections with the cLPL-specific antibody. LPL staining was robust on lectin-stained capillaries, but staining of cardiomyocytes was undetectable (Figure 1B and Supplemental Figure 2B).

**Figure 2:**
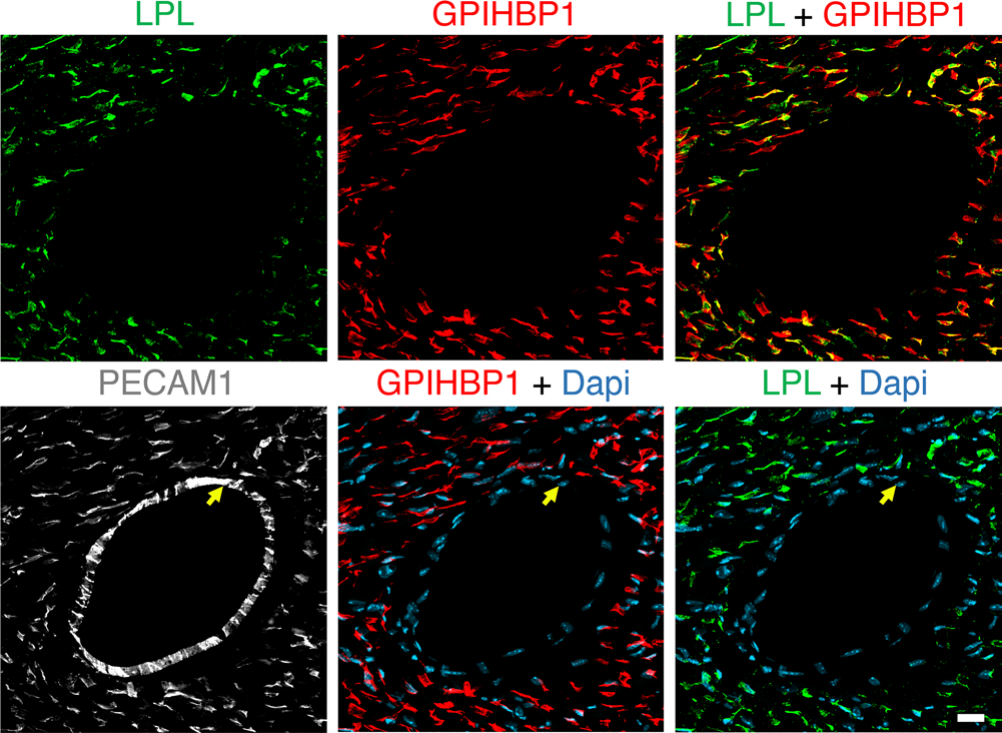
Confocal micrographs of LPL, GPIHBP1, and PECAM1 expression in mouse hearts. Heart sections were stained with antibodies against LPL, GPIHBP1, and PECAM1. Nuclei were stained with Dapi (blue). GPIHBP1 (red) and LPL (green) were detectable on ECs of capillaries but not on ECs of a large blood vessel (yellow arrow); PECAM1 (white) was found on ECs of both capillaries and the large blood vessels. Shown here are representative images from independent experiments with 3 mice. Scale bar: 20 μm.

**Figure 3:**
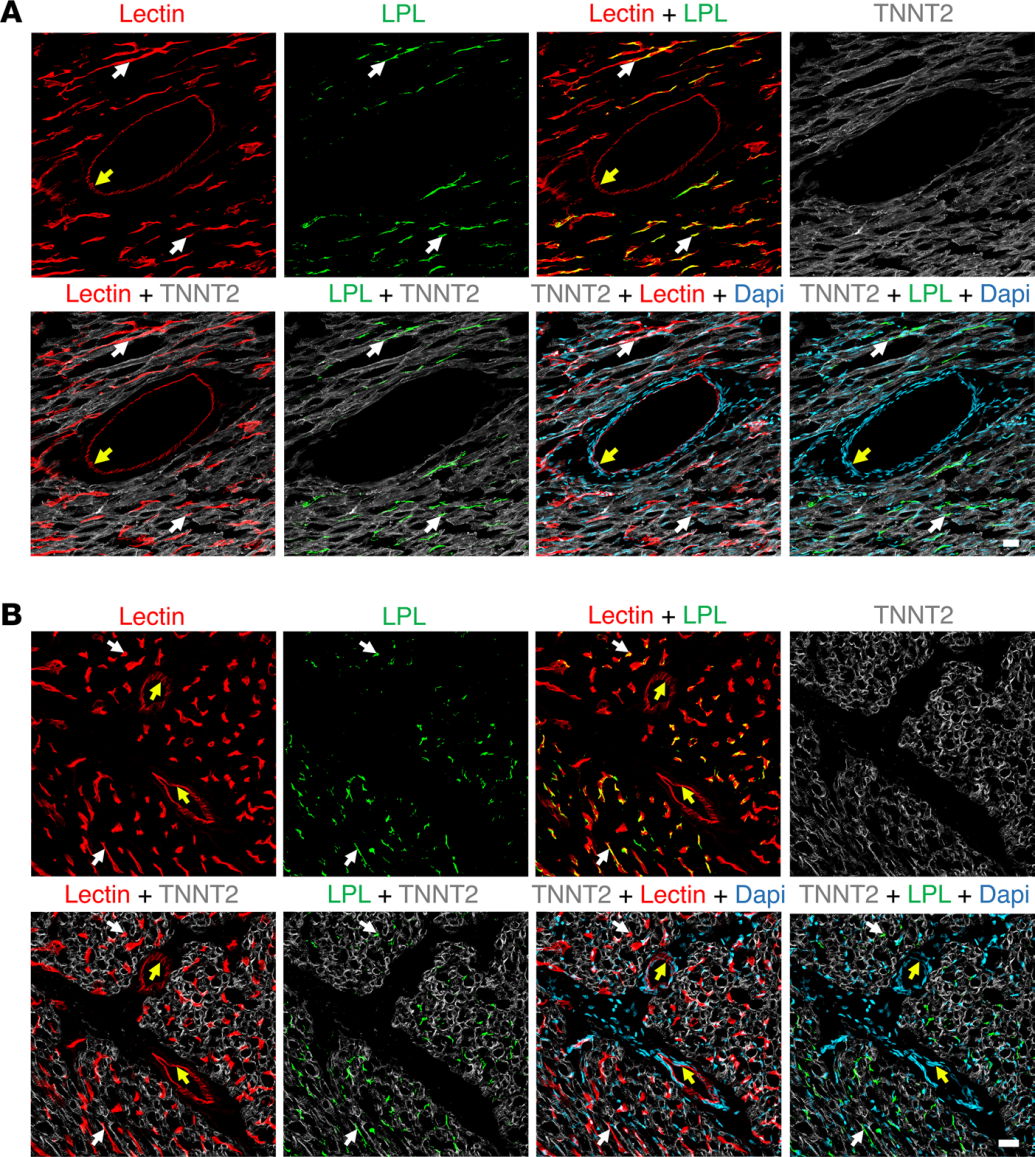
IHC studies of chicken heart showing that LPL is present on capillaries but not large blood vessels. (**A** and **B**) Heart sections were prepared from a chicken that had been injected intravenously with a fluorescein-labeled lectin (Lens culinaris agglutinin*,* red), which binds to glycoproteins on the luminal surface of blood vessels. Cryosections were stained with antibodies against chicken LPL (green) and TNNT2 (white). Confocal micrographs revealed LPL on chicken heart capillaries (white arrows) but not in a larger blood vessel (yellow arrow). Nuclei were stained with Dapi (blue). Shown here are representative images from independent experiments with 4 chickens. Scale bar: 20 μm.

To investigate mLPL distribution in different-sized blood vessels, we stained mouse heart sections with antibodies against PECAM1, LPL, and GPIHBP1. PECAM1 was present on ECs of capillaries and larger blood vessels, whereas LPL and GPIHBP1 were located only on capillary ECs (Figure 2). In the chicken heart cLPL was also located on capillaries but not larger blood vessels (Figure 3 and Supplemental Figure 3). In parallel studies, we gave chickens an intravenous injection of the fluorescein-labeled lectin and the cLPL-specific antibody. We observed binding of the cLPL antibody to the luminal surface of capillaries but not larger blood vessels (Supplemental Figure 4, A and B).

**Figure 4:**
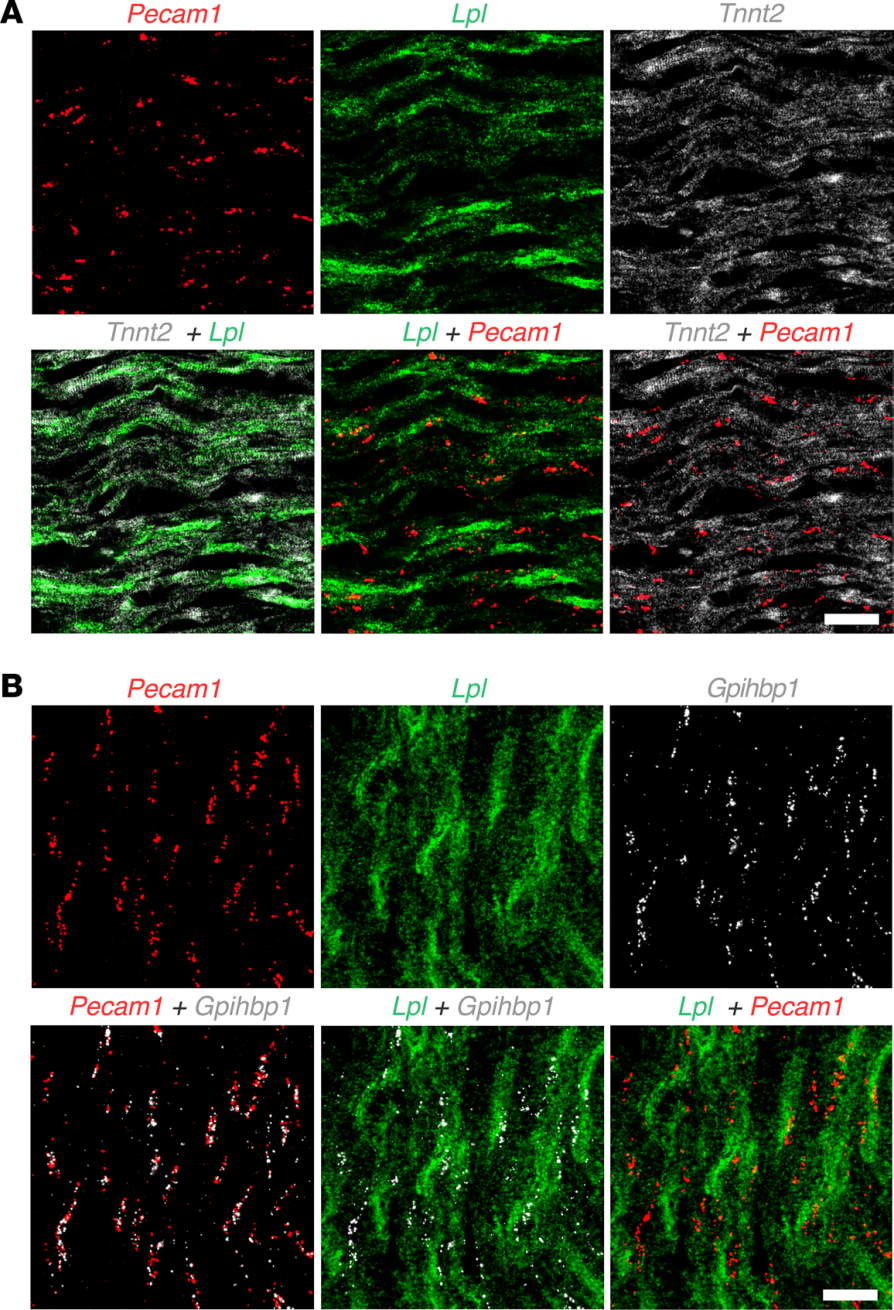
ISH studies on mouse heart with RNAscope probes for *Lpl, Tnnt2, Pecam1,* and *Gpihbp1*. (**A**) ISH studies of mouse heart, revealing abundant amounts of *Lpl* transcripts *(*green*)* and *Tnnt2* transcripts (encoding cardiac troponin T, white) in cardiomyocytes; transcripts for *Pecam1* (red) were in capillary ECs adjacent to cardiomyocytes. (**B**) ISH studies of mouse heart revealing abundant amounts of *Lpl* transcripts *(*green*)* in cardiomyocytes; *Pecam1* transcripts (red) and *Gpihbp1* transcripts (white) were in capillary ECs adjacent to cardiomyocytes. Shown here are representative images from 2 independent experiments. Scale bar: 20 μm.

**Figure 5:**
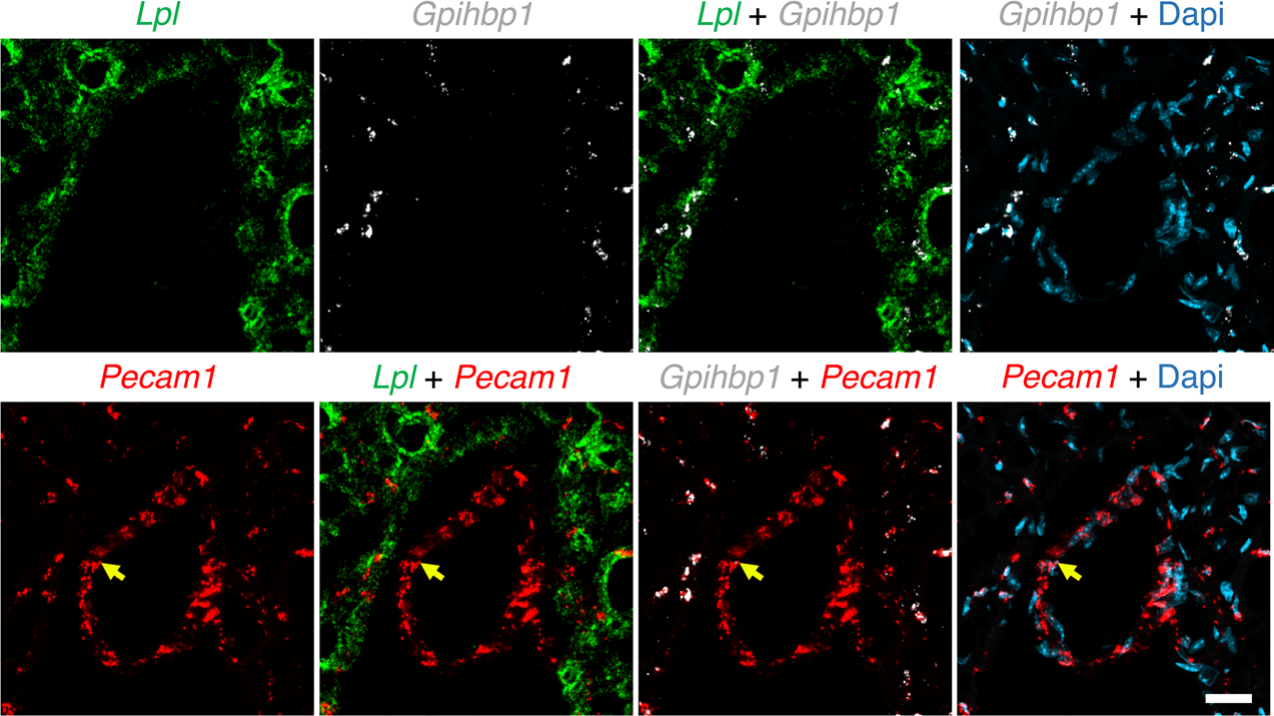
ISH studies of mouse heart with RNAscope probes for *Lpl*, *Gpihbp1*, and *Pecam1*. *Gpihbp1* (white) and *Pecam1* (red) transcripts were in capillary ECs adjacent to cardiomyocytes, which contained abundant *Lpl* transcripts (green). *Pecam1* transcripts, but not *Gpihbp1* transcripts, were located in ECs of a larger blood vessel (yellow arrow). Nuclei were stained with Dapi (blue). Shown here are representative images from independent experiments with 3 mice. Scale bar: 20 μm.

ISH studies of mouse hearts revealed that *Lpl* transcripts were extremely abundant in cardiomyocytes (with a signal intensity comparable to that of *Tnnt2*, the cardiac isoform of troponin) (Figure 4, A and B and Supplemental Figure 5A)*,* and *Pecam1* and *Gpihbp1* transcripts were in capillary ECs adjacent to cardiomyocytes (Figure 4, A and B and Supplemental Figure 5). In a combined ISH/IHC study, both *Gpihbp1* transcripts and GPIHBP1 protein were on capillaries adjacent to cardiomyocytes (which contained abundant *Lpl* transcripts) (Supplemental Figure 6). *Gpihbp1* transcripts were confined to capillary ECs, whereas *Pecam1* transcripts were in ECs of capillaries and larger blood vessels (Figure 5 and Supplemental Figures 7 and 8).

**Figure 6:**
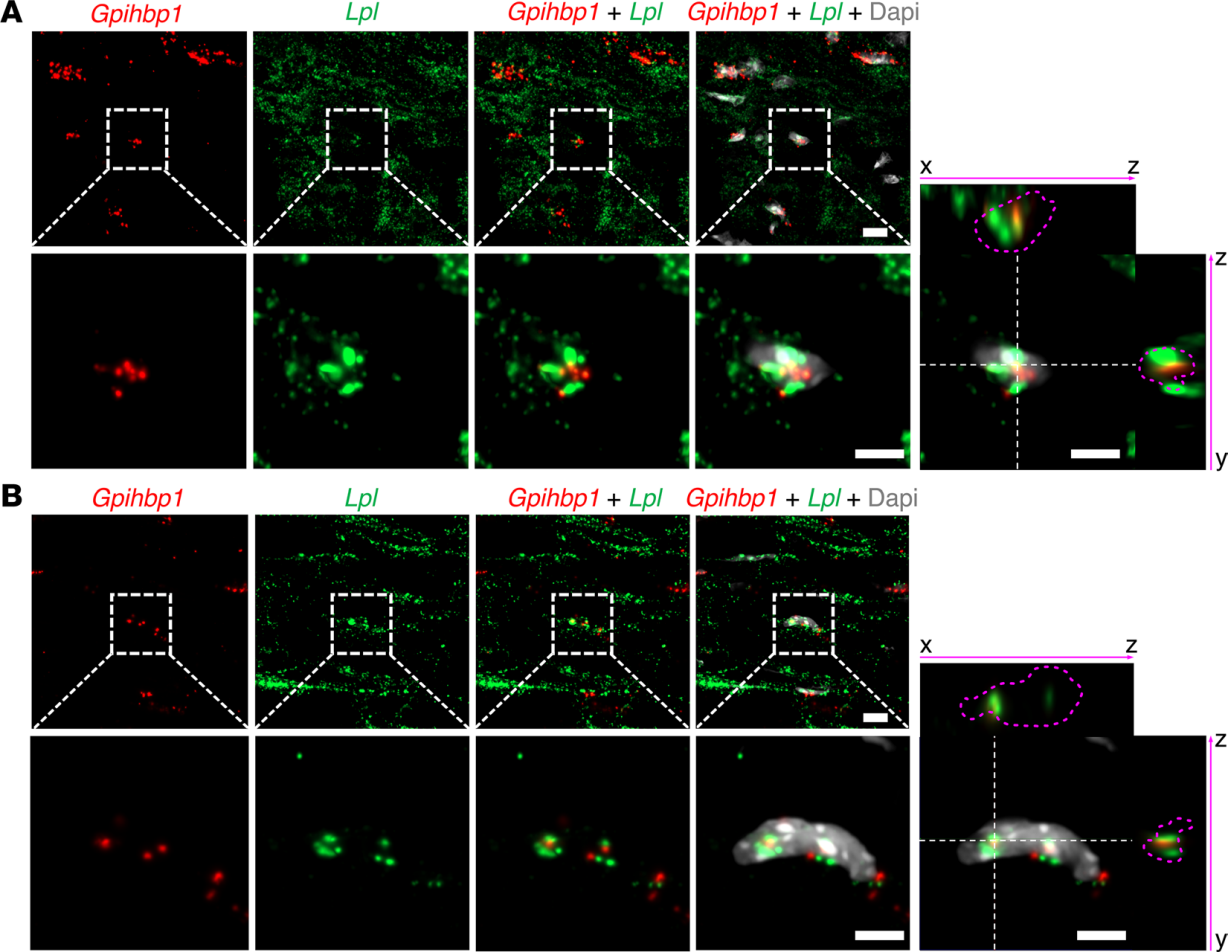
ISH studies of mouse heart with RNAscope probes for *Lpl* and *Gpihbp1*. (**A** and **B**) ISH studies of mouse heart demonstrated that *Lpl* (green) transcripts were abundant in cardiomyocytes, whereas *Gpihbp1* (red) transcripts were in capillary ECs adjacent to cardiomyocytes. Higher-magnification images of the boxed regions shows that a nucleus of a mouse heart capillary ECs contained both *Lpl* and *Gpihbp1* transcripts. Pink dashed lines indicate the border of the nucleus. Nuclei were stained with Dapi (white). Shown here are representative images from independent experiments with 2 mice. Scale bar: 5 μm.

Earlier studies reported that *Lpl* transcripts could be detected by reverse transcription PCR (RT-PCR) in freshly isolated ECs from mouse heart and brown adipose tissue (32, 33). Also, single cell RNA sequencing (scRNA-Seq) studies have detected *Lpl* transcripts in mouse heart capillary ECs (34). In our ISH experiments, we were able to find examples of *Lpl* transcripts in heart capillary ECs that contained *Gpihbp1* transcripts (Figure 6), but those examples were difficult to find, suggesting that *Lpl* transcript levels in mouse capillary ECs were low.

As experimental controls, we performed ISH studies in *Lpl*-deficient mice expressing human LPL transgenes. In hearts of *Lpl*-deficient mice harboring a human LPL transgene driven by the muscle-specific creatine kinase promoter (*Lpl* ^–/–^MCK–hLPL) (14, 35), human *LPL* transcripts were abundant in cardiomyocytes, while *Gpihbp1* and *Pecam1* transcripts were located in capillary ECs (Supplemental Figure 9, A and B). As expected, mouse *Lpl* transcripts were absent (Supplemental Figure 9C). In hearts of *Lpl*-deficient mice harboring a human *LPL* transgene driven by the Tie2 promoter (*Lpl* ^–/–^Tie2–hLPL) (14, 36), human *LPL* transcripts (along with *Gpihbp1* and *Pecam1* transcripts) were in capillary ECs (Supplemental Figure 10, A and B). As expected, mouse *Lpl* transcripts were absent (Supplemental Figure 10C).

**Figure 7:**
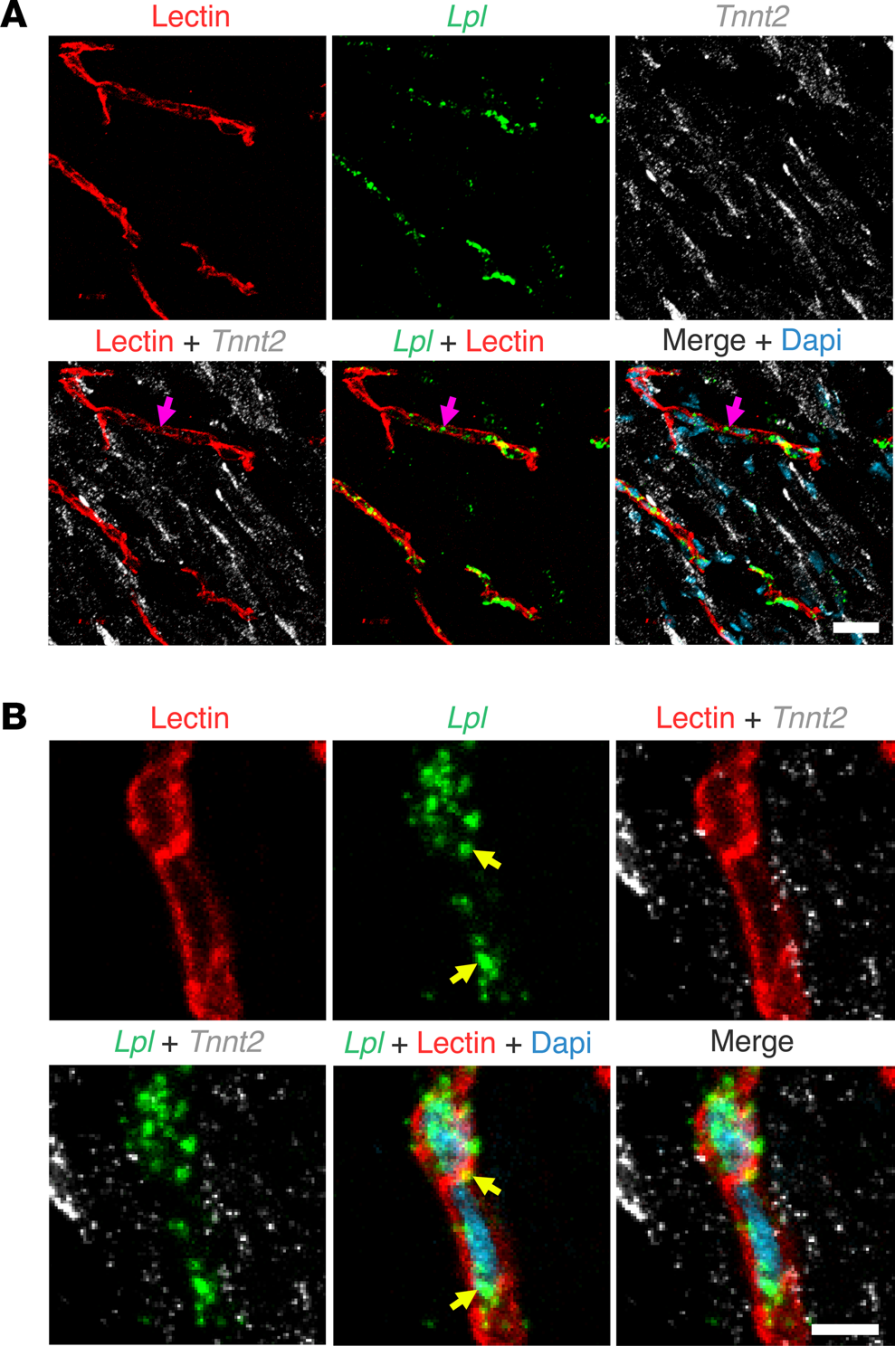
ISH studies on chicken heart with RNAscope probes for *Lpl* and *Tnnt2*. The chicken had been given an intravenous injection of a fluorescein-labeled lectin (Lens culinaris agglutinin*,* red) to stain the luminal surface of blood vessels. (**A**) ISH studies revealed abundant *Tnnt2* transcripts (white) in cardiomyocytes; *Lpl* transcripts (green) were abundant in capillary ECs (pink arrow). Scale bar: 5 μm. (**B**) *Lpl* transcripts (green) in chicken heart were found in capillary ECs, including in the cell nucleus (yellow arrows), but were not observed in adjacent *Tnnt2*-positive cardiomyocytes. Nuclei were stained with Dapi (blue). Shown here are representative images from independent experiments with 2 chickens. Scale bar: 5 μm.

**Figure 8:**
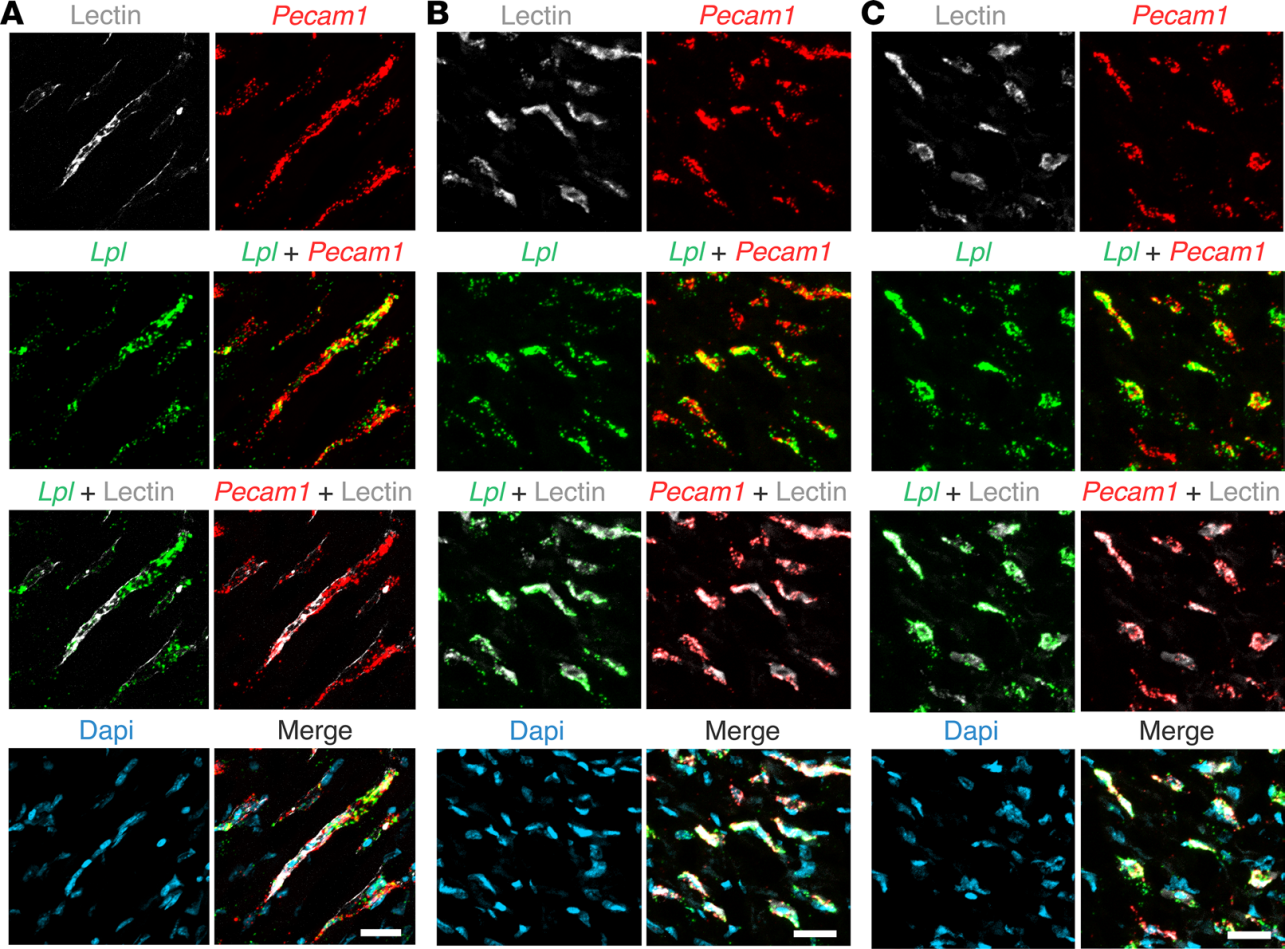
ISH studies of chicken heart with RNAscope probes against *Lpl* and *Pecam1*. The chicken had been given an intravenous injection of a fluorescein-labeled lectin (Lens culinaris agglutinin) to stain the luminal surface of blood vessels. (**A**–**C**) *Lpl* transcripts (green) were in capillary ECs, identified both by the fluorescein-labeled lectin (white) and by the presence of *Pecam1* transcripts (red). The *Lpl* signal outside of lectin-positive blood vessels was negligible. Shown are representative images from experiments with 4 chickens. Scale bar: 5 μm.

**Figure 9:**
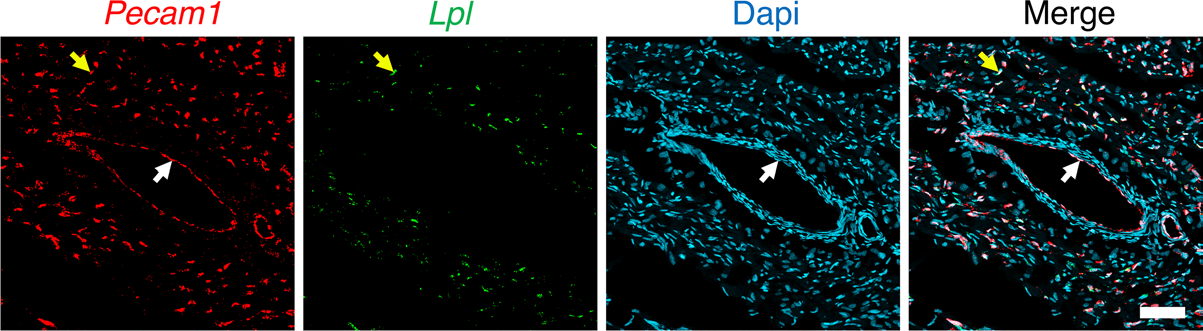
ISH studies of chicken heart with RNAscope probes against *Lpl* and *Pecam1*. *Pecam1* transcripts (red) were present in ECs of a large blood vessels (white arrow) and in a small capillary (yellow arrow). *Lpl* transcripts (green) were observed only in the capillary. Nuclei were stained with Dapi (blue). Shown is a representative image from experiments with 4 chickens. Scale bar: 50 μm.

To examine the location of *Lpl* transcripts in chicken hearts, we first marked the luminal surface of blood vessels with an intravenous injection of fluorescein-labeled lectin. *Lpl* transcripts were abundant in ECs of lectin-stained capillaries (Figure 7) but were negligible or absent in *Tnnt2*-positive cardiomyocytes (Figure 7). *Lpl* and *Pecam1* transcripts were both abundant in capillary ECs (Figure 8). *Lpl* transcripts were restricted to capillary ECs, whereas *Pecam1* transcripts were in ECs of both capillaries and larger blood vessels (Figure 9).

Our ISH studies suggested that *Lpl* transcripts were more abundant in chicken heart capillary ECs than in mouse heart capillary ECs, but the overall abundance of *Lpl* transcripts was greater in the mouse heart (due to an abundance of *Lpl* transcripts in cardiomyocytes). Quantitative RT-PCR (qRT-PCR) studies were consistent with that interpretation. We made PCR primers corresponding to perfectly conserved sequences of *Lpl* and *Gapdh* transcripts and used those primers to amplify a 236 bp *Lpl* cDNA fragment and a 125 bp *Gapdh* cDNA fragment from the RNA isolated from mouse and chicken hearts. The amount of the *Lpl* amplicon, relative to the *Gapdh* amplicon, was 22-fold higher in mouse heart than in chicken heart (Supplemental Figure 11). We also took advantage of mouse and chicken heart cDNA databases (37–39) to assess the rank order of *Tnnt2* and *Lpl* transcript abundance in mouse and chicken hearts. Those studies strongly suggested that *Lpl* transcripts were more abundant in the mouse heart. In the mouse heart on day 63, *Tnnc1* (troponin C1), *Actc1* (actin α cardiac muscle 1), *Myl2* (myosin light chain 2), and *Tnnt2* (troponin T2, cardiac type) transcripts ranked 18, 8, 11, and 33, respectively, in overall abundance; *Lpl* transcripts ranked 56 in abundance. In the chicken heart on day 7, *Tnnc1*, *Actc1*, *Myl2*, and *Tnnt2* transcripts ranked 25, 15, 11, and 16, respectively, in abundance; *Lpl* transcripts ranked far lower, at 389. At day 70, *Tnnc1*, *Actc1*, *Myl2*, and *Tnnt2* transcripts in the chicken heart ranked 26, 18, 16, and 14, respectively, in abundance, whereas *Lpl* transcript abundance ranked 430.

The distribution of chicken *Lpl* transcripts in pectoralis major and quadriceps was similar to the heart, with *Lpl* transcripts located in *Pecam1*-expressing capillary ECs (between *Tnnt3*-expressing myocytes) (Supplemental Figure 12). In the quadriceps of mice (which contains a mixture of red and white muscle fibers), *Lpl* transcripts were abundant in a subset of *Tnnt3*-expressing myocytes (Supplemental Figure 13A), consistent with the fact that LPL is expressed at high levels in muscles containing red muscle fibers but at very low levels in muscles containing white muscle fibers (40). *Lpl* transcripts were detected in *Pecam1*-positive capillary ECs (Supplemental Figure 13A) and *Pecam1*/*Gpihbp1*-positive capillary ECs (Supplemental Figure 13B). In quadriceps of *Lpl*^–/–^MCK–hLPL mice, human *LPL* transcripts were abundant in all skeletal myocytes, whereas *Pecam1* and *Gpihbp1* transcripts were in capillary ECs (Supplemental Figure 14A). In quadriceps of *Lpl*^–/–^Tie2–hLPL mice, human *LPL* transcripts, along with *Pecam1* and *Gpihbp1* transcripts, were in capillary ECs (Supplemental Figure 15A). As expected, mouse *Lpl* transcripts were absent in the quadriceps of *Lpl*^–/–^MCK–hLPL mice (Supplemental Figure 14B) and *Lpl*^–/–^Tie2–hLPL mice (Supplemental Figure 15B).

**Figure 10:**
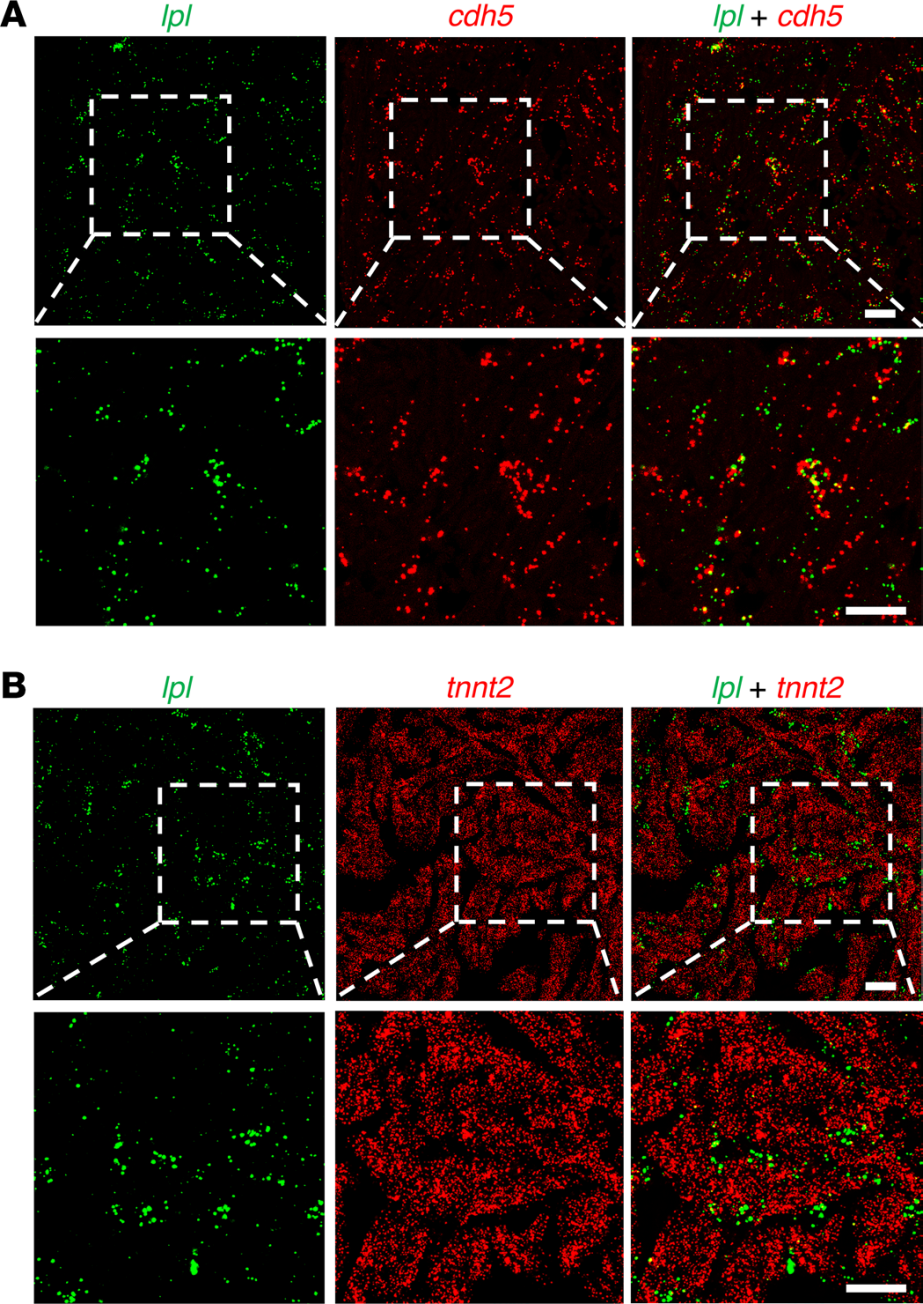
ISH studies on zebrafish heart with RNAscope probes. (**A**) Confocal micrograph revealing *lpl* (green) and *cdh5* (red) transcripts in ECs. (**B**) Confocal micrograph demonstrating that the distribution pattern of *lpl* and *tnnt*2 transcripts the zebrafish heart is distinct. *tnnt2* transcripts are in cardiomyocytes; *lpl* transcripts are in capillaries adjacent to cardiomyocytes. Boxed regions are shown below at a higher magnification. Shown are representative images from 4 zebrafish (2 males, 2 females). Scale bars: 20 μm.

**Figure 11:**
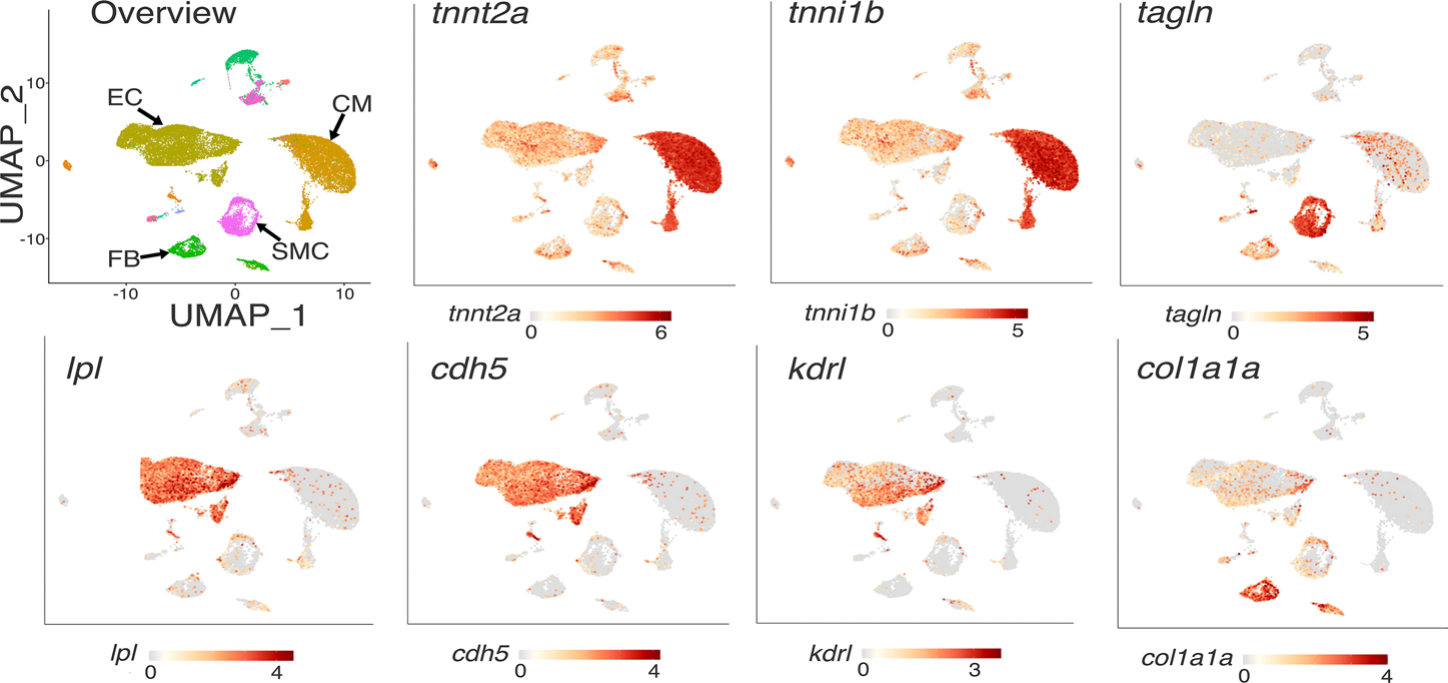
Single-cell RNA transcriptomic studies on zebrafish hearts, revealing LPL expression in the heart endothelial/endocardial cells. UMAP plot depicts the cellular composition of the zebrafish heart (*n* = 4 biologically independent samples), categorized into 4 major cell types. The expression patterns of 4 cell type–specific marker genes are shown (*cdh5* and *kdrl* for endothelial/endocardial cells [EC], *tnnt2a* and *tnni1b* for cardiomyocytes [CM], *tagln* for smooth muscle cells [SMC], *col1a1* for fibroblasts [FB]). The pattern of *lpl* expression resembles that for *cdh5* and *kdrl*.

We predicted that the pattern of *Lpl* transcript expression in chicken hearts — abundant in capillary ECs, absent in cardiomyocytes — might be shared by other lower vertebrates. This prediction was borne out. ISH studies of zebrafish heart revealed transcripts for both *lpl* and *cdh5* in ECs (*cdh5* encodes VE cadherin, an EC protein) (Figure 10A). The *lpl* transcripts were in capillaries adjacent to *tnnt2*-positive cardiomyocytes (Figure 10B). Consistent with these ISH findings, our analysis of a zebrafish heart scRNA-Seq database (41) revealed that *lpl* transcripts are abundant in *cdh5*- and *kdrl*-expressing ECs (Figure 11). Transcripts for *lpl* were detected in a very low percentage (2.4%) of cardiomyocytes (identified by expression of *Tnnt2* and *Tnni1b*). However, the *lpl*^+^ cardiomyocytes were enriched in many transcripts expressed at high levels in ECs, including EC-specific genes (e.g., *dll4*, *cdh5*, *sox7*, *sele*, *ramp2*, *dusp5*, and *fli1a*) (Supplemental Figure 16). The top 30 genes enriched in *lpl^+^* cardiomyocytes compared with *lpl^–^* cardiomyocytes (Wilcoxon rank-sum tests) are listed in Supplemental Figure 16. For each of those genes, the expression levels in the *lpl*^+^ versus. *lpl*^–^ cardiomyocytes were markedly different (*P* < 0.00001 after correction for multiple tests). These findings imply that the existence of *lpl* transcripts in the *lpl*^+^ cardiomyocytes did not actually reflect *lpl* expression in cardiomyocytes but instead reflected pieces of zebrafish cardiomyocytes attached to EC fragments.

**Figure 12:**
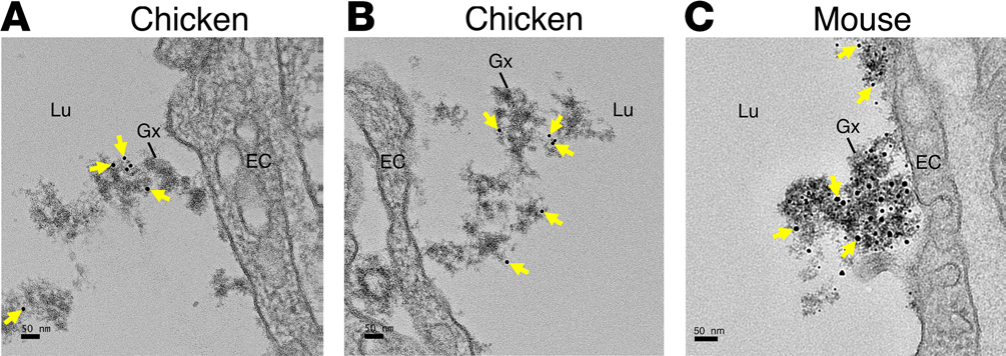
Transmission electron micrographs of mouse and chicken heart, revealing LPL in the glycocalyx of heart capillary ECs. (**A** and **B**) Electron micrographs of capillaries from a chicken heart that had been perfused with 10 nm gold nanobead–conjugated antibody against chicken LPL. (**C**) Transmission electron micrograph of a heart capillary from a mouse that had been injected with a 10-nm gold nanobead–conjugated rabbit antibody against mouse LPL. Yellow arrowheads point to gold nanobeads. The glycocalyx (Gx) was stained with LaCl_3_/DyCl_3_. Shown are representative images from experiments with 2 chicken hearts. Scale bar: 50 nm. Lu, lumen; EC, endothelial cell.

In the mouse heart, Song et al. (14) discovered that much of the LPL that GPIHBP1 transports into capillaries detaches from GPIHBP1 and is then captured within the HSPG-rich EC glycocalyx (as judged by confocal microscopy and immunogold electron microscopy). There was no binding of irrelevant control antibodies to the glycocalyx (14). We suspected that the LPL produced by chicken heart capillary ECs might also be detected in the EC glycocalyx. To test that idea, chickens were given an intravenous injection of the cLPL-specific antibody conjugated to 10 nm gold nanobeads, followed by staining of the glycocalyx with LaCl_3_/DyCl_3_ and fixation with glutaraldehyde. By transmission electron microscopy, gold nanobeads were observed in the chicken EC glycocalyx (Figure 12, A and B). In mice that had been injected with the mLPL-specific antibody conjugated to 10 nm gold nanobeads, we also observed gold nanobeads in the EC glycocalyx (Figure 12C).

**Figure 13:**
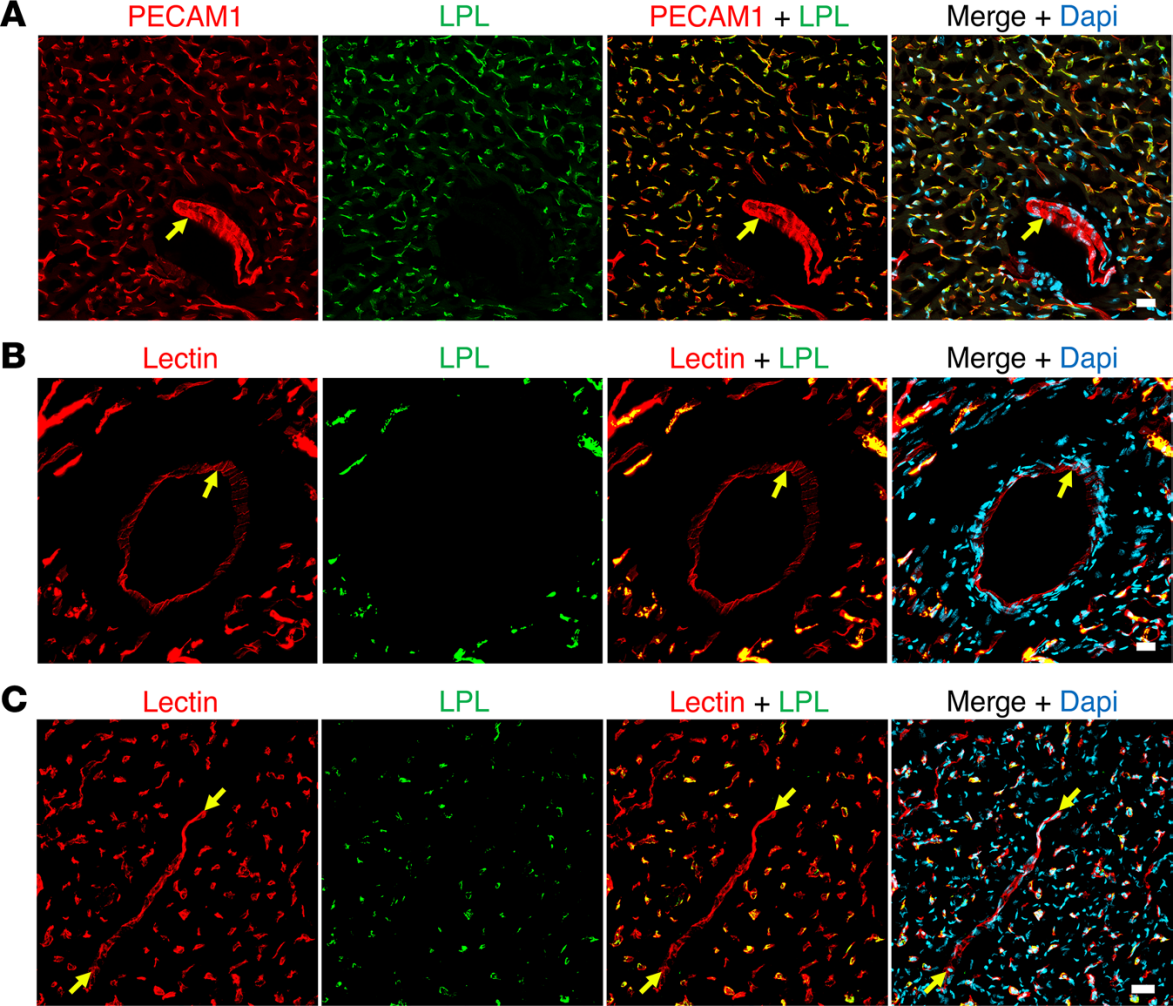
Recombinant human LPL, when injected intravenously into mice or chickens, binds to the luminal surface of heart capillaries. In mouse heart (**A**) and chicken hearts (**B** and **C**), recombinant human LPL (hLPL) binds avidly to ECs of capillaries but not larger blood vessels (yellow arrows). Shown are representative images from experiments with 3 chickens and 3 mice. Scale bar: 20 μm.

We examined the binding of recombinant hLPL to blood vessels of mouse and chicken hearts. When mice were given an intravenous injection of recombinant hLPL, we observed, by confocal immunofluorescence microscopy, hLPL binding to the capillary ECs but not to ECs of larger blood vessels (Figure 13A). When chickens were given an injection of hLPL, we also observed avid binding of the hLPL to capillary ECs but not to ECs of larger blood vessels (Figure 13, B and C).

## Discussion

We have long been puzzled by why GPIHBP1, the protein that moves LPL into capillaries in mammals and that is crucial for the lipolytic processing of TRLs, is absent in chickens and other lower vertebrates. The fact that an earlier study (8) observed LPL on chicken heart capillaries, despite the absence of GPIHBP1 (8), made the puzzle even more intriguing. To gain insights into LPL biology in chickens, we reasoned that it would be useful to compare the location of *Lpl* transcripts in mouse and chicken hearts. In the mouse heart, ISH studies with RNAscope probes revealed abundant *Lpl* transcripts in *Tnnt2*-expressing cardiomyocytes. *Lpl* transcripts could be detected in mouse heart capillary ECs but only in very low amounts. In the chicken heart, *Lpl* transcripts were quite abundant in capillary ECs but absent in cardiomyocytes. Consistent with those findings, LPL protein was easily detectable inside mouse cardiomyocytes but could not be detected in chicken cardiomyocytes. In the zebrafish heart, *lpl* transcripts were abundant in capillary ECs but absent in cardiomyocytes. These observations explain why GPIHBP1 is present in mammals but absent in lower vertebrates. Mammals require an LPL transporter because the LPL that is synthesized and secreted by cardiomyocytes needs to be moved from the interstitial spaces to the capillary lumen. Because LPL in the chicken heart is synthesized and secreted directly by capillary ECs, an LPL transporter in ECs is not required.

We are unaware of prior studies of LPL expression in hearts of chickens or other lower vertebrates; however, we were intrigued by a report of lipid-related gene expression in the pectoralis major of chickens with Wooden Breast disease, a myopathy that reduces the quality of breast meat and causes financial losses in the poultry industry (42). In that study, ISH studies of paraffin-embedded sections of pectoralis major from 3-week-old healthy and diseased chickens revealed *Lpl* transcripts in capillaries and veins (and in some cases arteries) (42). This study was important because it documented *Lpl* expression in blood vessels of skeletal muscle; however, the identification of specific cell types that expressed *Lpl* was limited by low-magnification images and the absence of cell type–specific ISH probes (42). In the current studies, we found that *Lpl* transcripts in chicken pectoralis major are abundant in *Pecam1*-expressing capillary ECs but absent in both myocytes and ECs of larger blood vessels.

We do not understand why mammals evolved a new strategy for plasma TG metabolism (i.e., abundant LPL expression in cardiomyocytes and an EC protein [GPIHBP1] that moves LPL into capillaries). A possible explanation is that the demand for LPL production is greater in mammals, requiring them to take advantage of the greater protein biosynthetic machinery of cardiomyocytes. Another possible explanation is that high levels of LPL expression in cardiomyocytes are required to keep up with increased LPL turnover. In mammals, the LPL in capillaries of oxidative tissues (e.g., heart skeletal muscle) is subjected to regulation by the ANGPTL3/ANGPTL8 complex (12, 43–46). After refeeding, plasma ANGPTL3/8 levels increase, resulting in increased inhibition of LPL activity (44, 46). ANGPTL3/8 also detaches LPL from the surface of cells (12, 47), explaining the reduced amounts of LPL in heart capillaries after feeding (43). Both the inhibition of LPL catalytic activity by ANGPTL3/8 and the detachment of LPL from the surface of cells are likely due to ANGPTL3/8-mediated unfolding of LPL’s amino-terminal hydrolase domain (12) (i.e., mirroring the mechanism by which ANGPTL4 regulates LPL activity (48–51)). We speculate that the robust ANGPTL3/8-mediated regulation of LPL in the heart capillaries of mammals could require them to produce large amounts of LPL in cardiomyocytes. Interestingly, ANGPTL8 — and thus the ANGPTL3/8 complex — is absent in chickens and other lower vertebrates (52). We speculate that the absence of ANGPTL3/8-mediated regulation of LPL in lower vertebrates could reduce LPL turnover and therefore obviate the need to produce large amounts of LPL by cardiomyocytes. Consistent with that reasoning, it is noteworthy that the rank order of *Lpl* transcript abundance in the chicken heart was far lower than the rank order for crucial sarcomere proteins (whereas in the mouse heart the rank order of *Lpl* transcript abundance was similar to the transcripts for sarcomere proteins).

While the cell types responsible for expressing *Lpl* transcripts in the mouse and chicken heart differ, two features of LPL biology are the same. One such feature is that the LPL in both species is confined to small capillaries (which almost certainly facilitates nutrient delivery to cardiomyocytes). In the mouse heart, the presence of LPL on capillary ECs involves three mechanisms. First, GPIHBP1 is expressed only in capillary ECs. By confocal microscopy, GPIHBP1 expression on ECs disappears as soon as the diameter of a capillary increases by approximately 50% (to become a minuscule venule). The distribution of LPL along capillaries closely mirrors the distribution of GPIHBP1 (11, 12). Second, LPL binds avidly and preferentially to capillaries. Catalytically active hLPL, when injected intravenously into mice, binds to the luminal surface of capillaries but not larger blood vessels (14). The mechanism for the preferential binding of recombinant LPL to capillary ECs but not larger blood vessels is not understood but presumably reflects heterogeneity in the protein and glycoprotein composition of the glycocalyx in different blood vessels. Third, *Lpl* transcripts are produced, albeit at very low levels, by capillary ECs of the mouse heart. In the chicken heart, LPL is also confined to capillary ECs because *Lpl* transcripts are made exclusively by chicken heart capillary ECs. Also, LPL binds avidly to chicken heart capillaries. When hLPL was injected intravenously into chickens, it bound to the luminal surface of capillaries but not larger blood vessels.

Another shared feature of LPL biology in mouse and chicken hearts is the presence of LPL in the glycocalyx of capillary ECs. Song et al. (14) discovered, by immunogold electron microscopy, that LPL is present within the glycocalyx of mouse heart capillary ECs. They also showed that glycocalyx-bound hLPL mediates TRL margination along heart capillaries and that it participates in the lipolytic processing of TRLs. In the current studies, we found, by immunogold electron microscopy, that LPL is detectable in the glycocalyx of heart capillary ECs in both chickens and mice.

Earlier studies showed that *Lpl* transcripts can be detected, by RT-PCR, in freshly isolated ECs from mouse heart and brown adipose tissue (32, 33). It appears, however, that the level of *Lpl* expression in mouse capillary ECs is insufficient to have major effects on plasma TG levels. First, an EC-specific *Lpl* knockout in mice had little or no effect on plasma TG levels (33). Second, plasma TG levels are markedly elevated (2,500–3,000 mg/dL) in *Gpihbp1*^–/–^ mice, implying that the LPL expression by ECs has minimal effects on plasma TG metabolism. In contrast to *Gpihbp1*^–/–^ mice, chickens maintain low plasma TG levels (25, 26). We suspect that the differences in plasma TG levels in chickens and *Gpihbp1*^–/–^ mice could relate, at least in part, to very different levels of *Lpl* expression in capillary ECs. In our ISH studies, *Lpl* transcripts were abundant in chicken heart capillary ECs, but *Lpl* transcripts in mouse capillary ECs were challenging to find.

While our studies were successful in identifying striking differences in LPL expression patterns in chicken and mouse hearts, they had limitations. For example, we only analyzed tissues of 5-day-old chickens. Also, our studies focused solely on *Lpl* expression in the heart (and to a lesser extent skeletal muscle). We did not study *Lpl* expression in other tissues, including adipose tissue. Ultimately, it will be important to define, by scRNA-Seq, *Lpl* expression in every cell type in multiple tissues, including heart, skeletal muscle, and adipose tissue.

In summary, *Lpl* is expressed at high levels in capillary ECs of the chicken heart, whereas in the mouse heart the LPL is expressed in abundant amounts in cardiomyocytes and then shuttled by GPIHBP1 to the capillary lumen. At this point, we can only speculate about why a new strategy for LPL-mediated intravascular lipolysis appeared in mammals. A possibility is that the robust production of LPL by cardiomyocytes in mammals relates, at least indirectly, to the importance of mammary glands and milk production in mammals. In the mammary gland, LPL is produced by parenchymal epithelial cells and adipocytes rather than by ECs (53). Another possibility is that high LPL turnover rates in mammals necessitated the production of abundant amounts of LPL in cardiomyocytes. Ultimately, a detailed understanding of the distinct strategies for intravascular TG metabolism in mammals and lower vertebrates will likely require a greater understanding of LPL synthesis and LPL turnover rates in multiple tissues, the biochemical and biophysical properties of LPL proteins in different species, the efficiency of LPL secretion by different cell types, and the TG utilization rates in different tissues.

## Methods

### Sex as a biological variable.

Male and female mice express GPIHBP1 and LPL and carry out the intravascular processing of lipoproteins. We showed that male and female WT mice, *Gpihbp1*^–/–^ mice, and *Gpihbp1*^–/–^ mice carrying a human *LPL* transgene have very similar plasma TG levels (12–15). In the current study, we used predominantly male mice; female mice were used in Figure 4 (which as expected revealed similar levels of *Lpl, Gpihbp1,* and *Pecam1* transcripts in the heart). We used 5-day-old male and female chicks, a time point when it is not possible to reliably distinguish between male and female chickens. We examined equal numbers of male and female zebrafish.

### Mouse and chicken studies.

C57BL/6J mice were purchased from The Jackson Laboratory and fed a chow diet. *Lpl* ^–/–^MCK–hLPL and *Lpl* ^–/–^Tie2–hLPL mice have been described previously (14, 35, 36). Studies were performed in 8–9 week-old male and female mice weighing approximately 30 g. Five-day-old Marans chickens (mean weight, 60 g) were provided by UCLA’s Division of Laboratory Animal Medicine.

### Antibodies.

Chicken LPL was expressed in Drosophila *S2* cells (31); the recombinant protein was used to generate a rabbit polyclonal antibody against chicken LPL (antibody 4727). A rabbit polyclonal antibody against mouse LPL (antibody 3174), created with the same approach, was described previously (12–14). Antibodies were purified from rabbit serum on protein G–Sepharose columns. A mouse monoclonal antibody (mAb) against troponin T was from Thermo Fisher Scientific (MA5-12960); a hamster mAb against PECAM1 (antibody 2H8) was produced from a hybridoma cell line from the Developmental Studies Hybridoma Bank at the University of Iowa; a rat mAb against mouse GPIHBP1 (antibody 11A12) (11) and a human LPL–specific rabbit polyclonal antibody (antibody 1256) have been described previously (54). Alexa Fluor–conjugated secondary antibodies were purchased from Thermo Fisher Scientific (A-21202, A-10037, A-31571, A-21206, A-10042, A-31573).

### Mouse and chicken experiments.

Mice were anesthetized with isoflurane, and 300 μg of Alexa Fluor 488–conjugated mAb 2H8 (against PECAM1) was injected via the tail vein. After 2 minutes, the thoracic cavity was opened, and the heart was perfused with 30 mL of PBS through the left ventricle, followed by 10 mL of 3% paraformaldehyde (PFA). Hearts were collected and embedded in OCT medium on dry ice and stored at –80°C. Chickens were anesthetized with a mixture of 20 μg/kg dexomitor, 25 mg/kg ketamine, and 2 mg/kg midazolam for chickens weighing > 75 g or a mixture of 10 μg/kg dexomitor, 12.5 mg/kg ketamine, and 1.0 mg/kg midazolam for chickens weighing < 75 g. Fluorescein-labeled *Lens culinaris* *agglutinin* (500 μg) (Vector Laboratories) was injected via the brachial vein alone or in combination with 500 μg of the chicken LPL–specific antibody 4727 and/or 500 μg of hamster nonimmune antibody (as a perfusion control). After 4 minutes, the heart was perfused with 50 mL of PBS through the left ventricle, followed by 30 mL of 3% PFA in PBS. Hearts were collected and fixed in 3% PFA at 4°C for 1 hour. Tissues were embedded in OCT on dry ice and stored at –80°C.

### IHC studies.

For IHC studies of the mouse heart, 10 μm–thick frozen sections were fixed in ice-cold methanol for 20 minutes, washed in PBS (Mg^2+^/Ca^2+^), and permeabilized with 0.2% Triton X-100 in PBS. Sections were incubated for 1 hour at room temperature in blocking buffer (PBS containing 0.2% BSA and 5% donkey serum) and then overnight at 4°C with primary antibodies. On the next day, after washing in blocking buffer, the sections were incubated with Alexa Fluor–conjugated secondary antibodies for 45 minutes at room temperature. Sections were postfixed with 3% PFA for 5 minutes, and cell nuclei were stained with Dapi (5 μg/mL) for 5 minutes. Slides were mounted with prolong gold antifade (Thermo Fisher Scientific). Images were recorded on an LSM980 microscope (Zeiss) with 20 × or 63 × objectives. Antibodies were used at the following concentrations: mAb 11A12, 10 μg/mL; rabbit antibody 3174, 7.5 μg/mL; rabbit polyclonal antibody 4727, 10 μg/mL; the mAb against TNNT2 (Thermo Fisher Scientific), 7.5 μg/ml. Alexa Fluor–conjugated secondary antibodies were used at a dilution of 1:200. For experiments with the TNNT2-specific antibody, cryosections of mouse heart were pretreated with the Mouse-on-Mouse kit (Vector Laboratories).

### Studies of isolated perfused chicken hearts.

Hearts were removed from the thoracic cavity of anesthetized 5-day-old chickens, cannulated, and submerged in Tyrode’s buffer (136 mM NaCl, 5.4 mM KCl, 0.33 mM NaH_2_PO_4_, 1 mM MgCl_2_, 10 mM HEPES, pH 7.4, 10 mM glucose). Hearts was perfused with 2 mL of Tyrode’s buffer containing 200 μg of fluorescein-labeled Lens culinaris agglutinin and 500 μg of recombinant human LPL (55). After 4 minutes, the heart was perfused with 4 mL of Tyrode’s buffer, followed by 3 mL of 3% PFA. Hearts were fixed in 3% PFA at 4°C for 1 hour and then embedded in OCT compound. Sections were stained with the rabbit antibody against human LPL (1256). Images were obtained on an LSM980 microscope (Zeiss).

### Western blots.

CHO-K1 cells were transfected with V5-tagged expression vectors for chicken, mouse, and human LPL. After 24 hours, the cells were harvested and resuspended in 200 μL of RIPA buffer (50 mM Tris pH 7.4, 150 mM NaCl, 1 mM EDTA, 1% NP40, 0.25% sodium deoxycholate, 1 mM PMSF, 1 mM NaF), supplemented with a protease inhibitor cocktail [cOmplete ULTRA EDTA-free, Roche]) and then sonicated. The lysates were centrifuged at 18,000*g* in a microcentrifuge at 4°C for 15 minutes; supernatants were collected; the proteins were size-fractionated on 12% Bis-Tris SDS-PAGE gels and transferred to a nitrocellulose membrane. The membranes were incubated with the rabbit antibody against chicken LPL (4727; 10 μg/mL), followed by an incubation with IRDye680-conjugated donkey antibody against rabbit IgG (1:2,000; Li-COR, 926-68073). After washing with PBS Tween (0.1%), the membrane was incubated with a mouse mAb against the V5 tag (10 μg/mL; Thermo Fisher Scientific, R960-25), followed by an incubation with IRDye800-conjugated donkey antibody against mouse IgG (1:2,000; Li-COR, 926-32212). Signals were detected with an Odyssey infrared scanner (LI-COR).

### Gene expression studies.

RNA was extracted from flash-frozen tissues with TRI reagent (Molecular Research), and cDNA was prepared with random primers, oligo(dT), and SuperScript III (Invitrogen). Real-time PCR was performed in triplicate with a QuantStudio 5 Real-Time PCR System (Applied Biosystems) and a SYBR Green PCR Master Mix. We amplified segments of mouse and chicken *Lpl* and *Gapdh* transcripts with oligonucleotide primers (Supplemental Table 1) corresponding to perfectly conserved sequences in the mouse and chicken transcripts. We also analyzed the rank order of transcript abundance in mouse and chicken heart expression databases (38, 39). Transcript abundance rankings were based on transcripts per million (TPM) (37).

### ISH studies.

ISH studies on mouse and chicken tissues were performed with the RNAscope Multiplex Fluorescent Detection Kit v2.0 (ACDBio). Paired double-Z oligonucleotide probes were designed and manufactured by ACDBio. 10 μm–thick sections of PFA-fixed and OCT-embedded tissues were incubated in 3% PFA for 1 hour (in mice) or 2 hours (in chickens that had not been injected with the fluorescein-labeled lectin) or 6 hours (in chickens that had been injected with the fluorescein-labeled lectin) at 4°C, followed by a 10 minute incubation with hydrogen peroxide at room temperature. A protease IV incubation step (for mice and chickens that were not injected with the fluorescein-labeled lectin) and a protease III incubation step (for chickens that had been injected with the fluorescein-labeled lectin) were carried out for 30 minutes at room temperature, followed by hybridization of the probes for 2 hours at 40°C in a HybEZ II Oven. Signals were amplified with 3 consecutive amplification steps and detected with Vivid fluorophores 520, 570, or 650. Slides were counterstained with Dapi. RNA integrity was tested with negative and positive control probes from ACDBio. Confocal images were recorded with an LSM980 microscope (Zeiss).

In some experiments, ISH and IHC procedures were coupled. Tissue sections were incubated in 4% PFA for 15 minutes at room temperature and then blocked in 5% donkey serum in PBS for 1 hour at room temperature. Subsequently, sections were treated with hydrogen peroxide for 10 minutes at room temperature and then incubated overnight at 4°C with GPIHBP1 mAb (11A12) and TNNT2 mAb (Thermo Fisher Scientific, MA5-12960) adjusted to 20 μg/mL in codetection antibody diluent. On the next day, sections were treated with protease III for 30 minutes at room temperature. ISH steps were carried out as described earlier. After completing the ISH steps, sections were incubated with Alexa Fluor–conjugated secondary antibodies (Thermo Fisher Scientific, A-21202, A-10037, A-31571, A-21206, A-10042, A-31573; diluted 1:200 in codetection diluent) for 30 minutes at room temperature. After washing in PBS containing 0.2% Tween, sections were counterstained with Dapi. Images were recorded on an LSM980 microscope (Zeiss).

Six-month-old zebrafish were anesthetized in 0.01% (w/v) Tricane (Sigma Aldrich) for 10 minutes, followed by a 10 minute incubation in an ice bath. Hearts were isolated and fixed in 4% PFA for 4 hours at 4°C. Hearts were immersed overnight in 20% sucrose followed by 30% sucrose and NEG-5 (Richard Allan Scientific), and then embedded in a dry ice/isopentane slurry. RNAscope Multiplex Fluorescent Reagent Kit (v.2) (ACDBio) and TSA Plus reagents (Perkin Elmer) were used according to the manufacturer’s instructions. Briefly, 14 μm cryosections were dehydrated for 5 minutes with ethanol (50%, 70%, and twice with 100%) at room temperature, and the slides were stored overnight in 100% ethanol. Sections were air dried, and a hydrophobic barrier was created around the section with Immedge Hydrophobic Barrier Pen (Vector Laboratory). Sections were treated with hydrogen peroxide for 10 minutes at room temperature, rinsed twice with water, and permeabilized for 20 minutes with protease III or protease IV. After rinsing twice with PBS, probe mixtures (100 μL) were applied to the sections and incubated for 2 hours at 40°C. Fluorescent signals were developed and amplified according to the manufacturer’s instructions. Images were acquired on a Leica SP8 confocal microscope with a 63 × objective.

### Immunogold electron microscopy.

Immunogold electron microscopy procedures for the mouse heart with the mouse LPL–specific antibody 3174 were performed as described (14). Chicken hearts were collected from anesthetized 5-day-old chickens and perfused with 1 mL of a saline/HEPES buffer (0.9% NaCl in 10 mM HEPES, pH 7.4) via the right ventricle, followed by perfusion with 2 mL of the saline/HEPES buffer via the left ventricle. Hearts were cannulated, flushed with 1 mL of saline/HEPES buffer, and submerged in 30 mL of the saline/HEPES buffer. Subsequently, the cannulated heart was perfused with 1 mL saline/HEPES containing 300 μg of a 10 nm gold nanobead–labeled antibody against chicken LPL (4727). Next, the cannulated heart was perfused with 5 mL of saline/HEPES (pH 7.2) containing 0.5% LaCl_3_/DyCl_3_ (Sigma Aldrich), followed by 10 mL of saline/HEPES (pH 7.2) containing 0.5% LaCl_3_/DyCl_3_ and 2.5% (vol/vol) of glutaraldehyde (Electron Microscopy Sciences). Next, hearts were incubated in saline/HEPES (pH 7.2) containing 0.5% LaCl_3_/DyCl_3_ and 2.5% (vol/vol) glutaraldehyde at 4°C for 1 hour. Sections were prepared for transmission electron microscopy as described (56).

### Analyzing scRNA-Seq data from zebrafish hearts.

Zebrafish heart scRNA-Seq data was obtained from NCBI GEO database under accession number GSE159032. We analyzed data from 4 healthy control zebrafish hearts (41). The raw counts data were processed in R Seurat packages (v. 4.3.0) for quality control, normalization, dimensional reduction, differential expression analyses, and visualizations. To integrate the data from different samples in the UMAP visualization, the RPCA method in the Seurat package was used. The Seurat FindMarkers function was used to compare gene expression differences between the *lpl^+^* cardiomyocytes and *lpl^–^* cardiomyocytes, which applies a Wilcoxon Rank Sum test between the 2 cardiomyocyte groups and performs multiple test corrections with the Bonferroni method.

### Statistics.

For statistical analyses of *Lpl* transcript levels, normalized to *Gapdh* transcript levels, in chicken and mouse hearts, we applied a 2-tailed Student’s *t* test (see Supplemental Figure 11). To assess the significance of differentially expressed genes in *lpl*^+^ versus. *lpl*^–^ zebrafish cardiomyocytes (see Supplemental Figure 16), we used a Wilcoxon Rank Sum test and multiple test corrections with the Bonferroni method.

### Study approval.

All mouse and chicken studies were approved by the UCLA’s Animal Research Committee according to guidelines in the NIH Guide for the Care and Use of Laboratory Animals. Zebrafish experiments were carried out in the Uppsala University Zebrafish Facility according to standard procedures (57) with approval from the Swedish Board of Agriculture (5.8.18-06282/2023).

### Data availability and sharing of reagents.

Supporting data for Supplemental Figures 11 and 16 are available in the Supplemental Data Values file. Raw data for confocal micrographs will be made available upon request. All materials and methods used in the study will be made available to researchers for their own use.

Author contributions

LPN, CB, MAM, LH, APB, LGF, and SGY designed research. LPN, TAW, WS, YY, HJ, YT, PHK JRK, KX, RGY, APT, JS, AMP, MP, GB, HA, KK, MAM, CB, LH, JLG, APB, and LGF performed research. LPN, TAW, YT, APT, LH, CB, MAM, APB, LGF, and SGY analyzed data. LPN, CB, MAM, APB, LGF, and SGY wrote the paper. The order of each author in the list was determined by SGY and was open for discussion.

## Supplementary Material

Supplemental data

Unedited blot and gel images

Supporting data values

## Version 1 10/22/2024

Electronic publication
