# Supplementary material for: Distinct strategies for intravascular triglyceride metabolism in hearts of mammals and lower vertebrate species
Source: JCI Insight. 2024 Sep 17;9(20):e184940. doi: 10.1172/jci.insight.184940 (PMC11529983; doi:10.1172/jci.insight.184940)
Supplement: Supplemental data [file jciinsight-9-184940-s172.pdf]

Supplementary Figures and Table for:

Distinct strategies for intravascular triglyceride metabolism in hearts of mammals and lower vertebrate species

Le Phuong Nguyen[1], Wenxin Song[1], Ye Yang[1,2], Anh P. Tran[1], Thomas Weston[1], Hyesoo Jung[1], Yiping Tu[1], Paul H. Kim[1], Joonyoung R. Kim[1], Katherine Xie[1], Rachel G. Yu[1], Julia Scheihauer[1], Ashley M. Presnell[1], Michael Ploug[3,4], Gabriel Birrane[5], Jeffrey L. Goodwin[6], Hannah Arnold[7], Katarzyna Koltowska[7,8], Maarja Andaloussi Mäe[7], Liqun He[7], Christer Betsholtz[7,9], Anne P. Beigneux[1], Loren G. Fong[1,†], and Stephen G. Young[1,2,†]

[1]Department of Medicine and [2]Human Genetics, David Geffen School of Medicine, UCLA, Los Angeles, California, USA; [3]Finsen Laboratory, Copenhagen University Hospital-Rigshospitalet, DK-2200 Copenhagen N, Denmark; [4]Finsen Laboratory, Biotech Research and Innovation Centre, University of Copenhagen, DK-2200 Copenhagen N, Denmark; [5]Division of Experimental Medicine, Beth Israel Deaconess Medical Center, Boston, MA 02215; [6]Department of Immunology, Genetics, and Pathology, Rudbeck Laboratory, Uppsala University, Uppsala, Sweden; [7]Department of Medicine-Huddinge, Karolinska Institute Campus Flemingsberg, Huddinge, Sweden; [8]Division of Laboratory Animal Medicine, David Geffen School of Medicine, UCLA, Los Angeles, California, USA

Supplementary Figures and Figure Legends

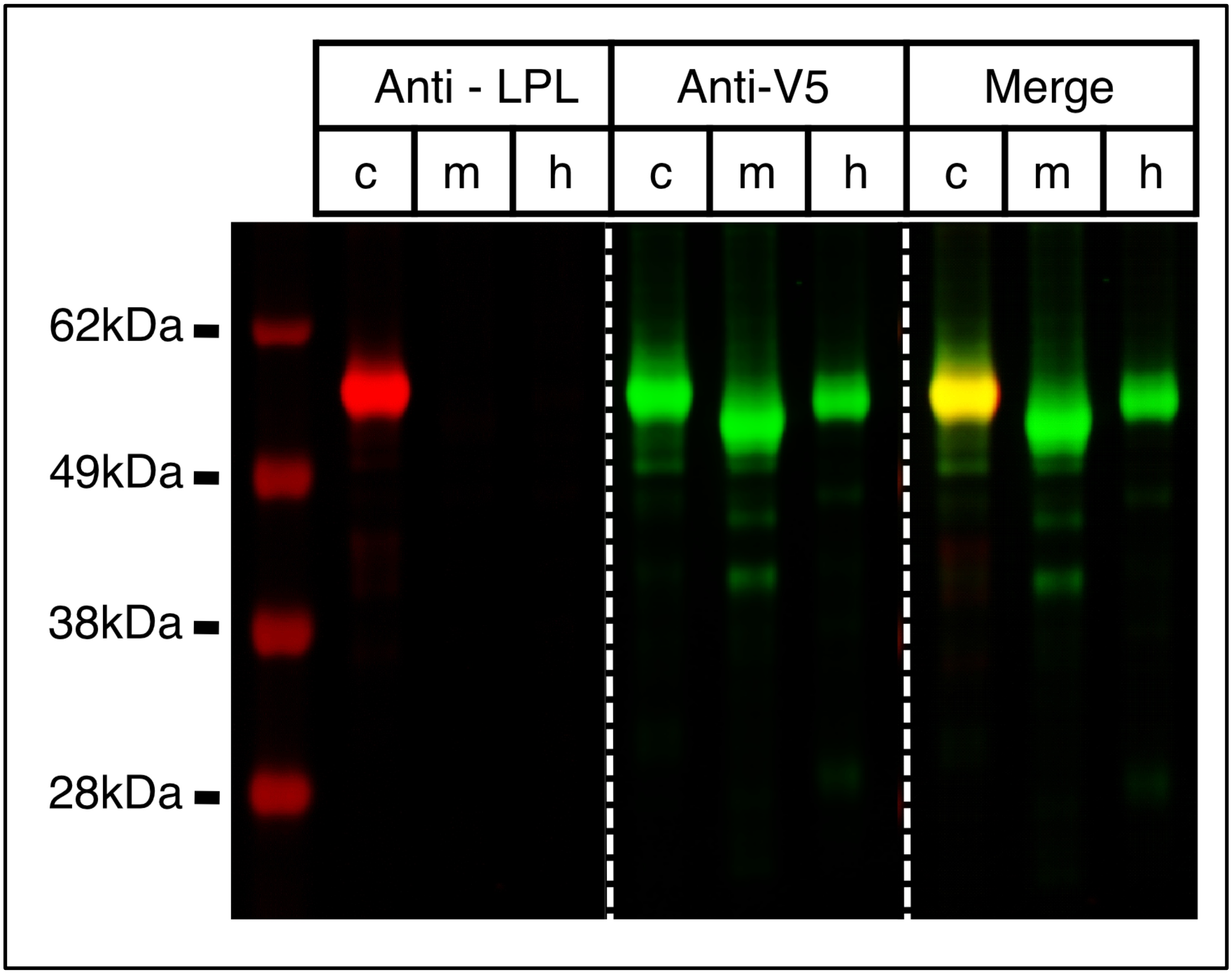

Supplemental Figure 1. Western blot testing the ability of the rabbit antibody against chicken LPL to bind to chicken, mouse, and human LPL. CHO-K1 cells were transfected with V5-tagged expression vectors for chicken (c), mouse (m), or human (h) LPL. After 24 h, the cells were collected; the proteins were size fractionated by SDS-polyacrylamide gel electrophoresis and transferred to nitrocellulose membranes. Western blots were performed with the rabbit polyclonal antibody against chicken LPL and a mouse monoclonal antibody against the V5 epitope tag. Binding of antibodies was detected with an IRDye680-labeled goat antibody against rabbit IgG and an IRDye800-labeled goat antibody against mouse IgG.

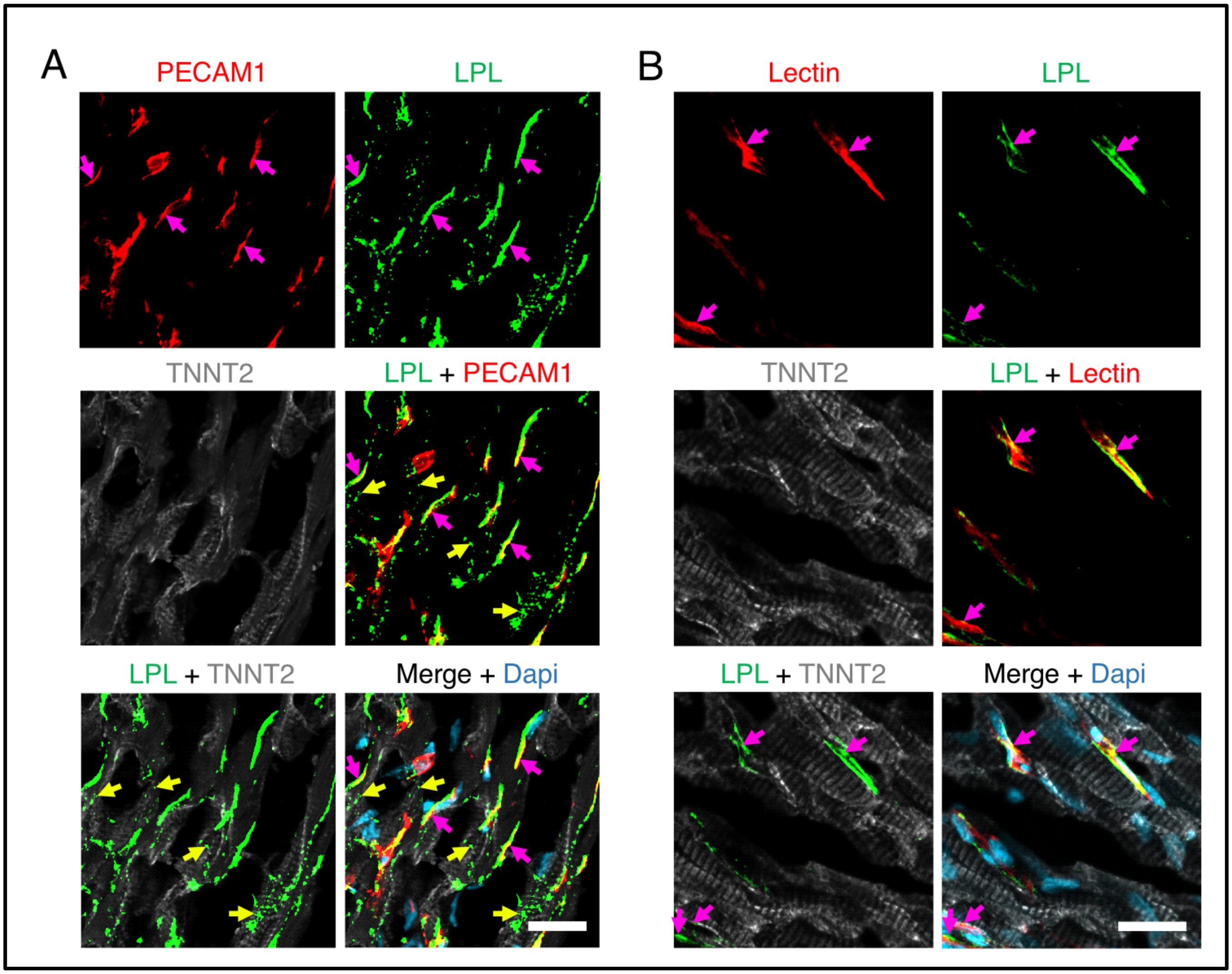

Supplemental Figure 2. Confocal micrographs of immunohistochemistry studies showing that LPL is located on capillaries of mouse and chicken hearts. Cryosections of mouse and chicken heart tissue were stained with antibodies against cardiac troponin T (TNNT2, *white*) and either a mouse LPL–specific antibody (3174) or a chicken LPL–specific antibody (4727). Nuclei were stained with Dapi (*blue*). (**A**) Heart section from a mouse that had been injected intravenously with an Alexa Fluor 488–labeled PECAM1-specific mAb (2H8, *red*). Mouse LPL (*green*) was detected on PECAM1-positive capillary ECs (*pink* arrows) and inside cardiomyocytes (*yellow* arrows). (**B**) Heart section from a chicken that had been injected intravenously with a fluorescein-labeled lectin (*Lens culinaris agglutinin*, *red*). Chicken LPL (*green*) was detected on capillary ECs (*pink* arrows); LPL inside cardiomyocytes was negligible or absent. Scale bar, 20 µm.

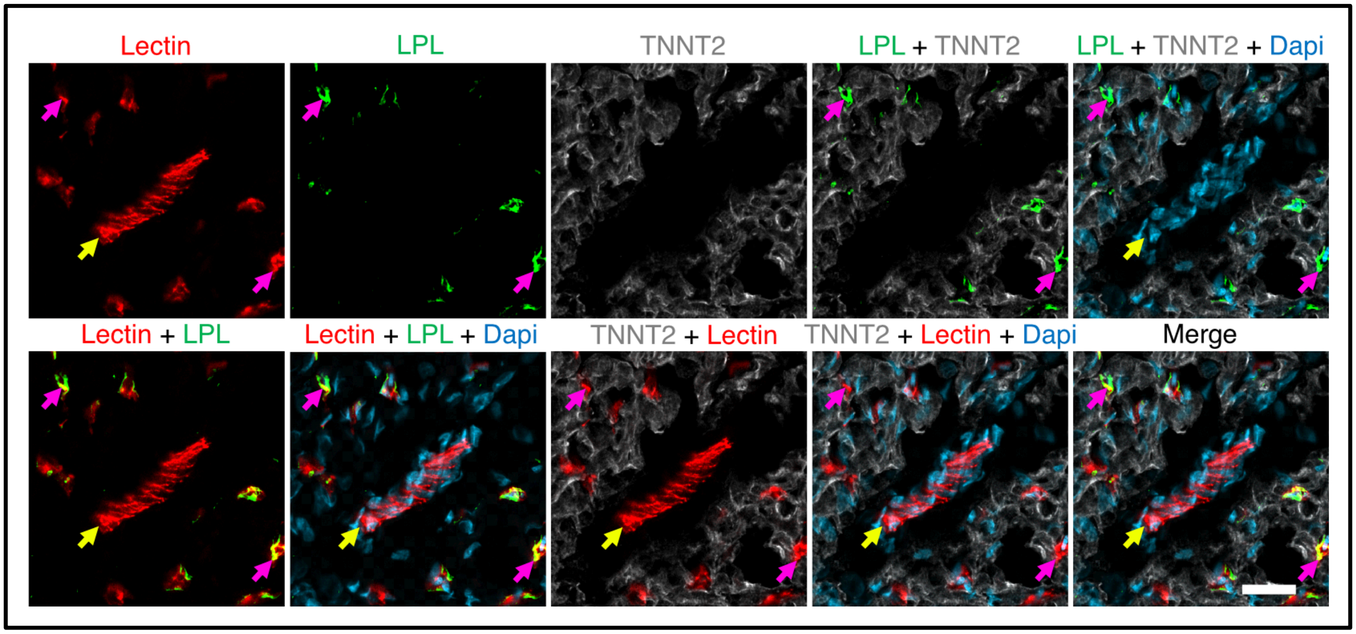

Supplemental Figure 3. Immunohistochemistry studies reveal LPL in chicken heart capillaries but not in a larger blood vessel. Heart sections were prepared from a 5-day-old chicken that had been injected intravenously with fluorescein-labeled lectin (*red*) to stain blood vessels. Cryosections were stained with antibodies against chicken LPL (*green*) and TNNT2 (*white*). Confocal micrographs show LPL in capillaries (*pink* arrows) but not in the larger blood vessel (*yellow* arrow). LPL was nearly undetectable in TNNT2-positive cardiomyocytes. Nuclei were stained with Dapi (*blue*). Scale bar, 20 µm.

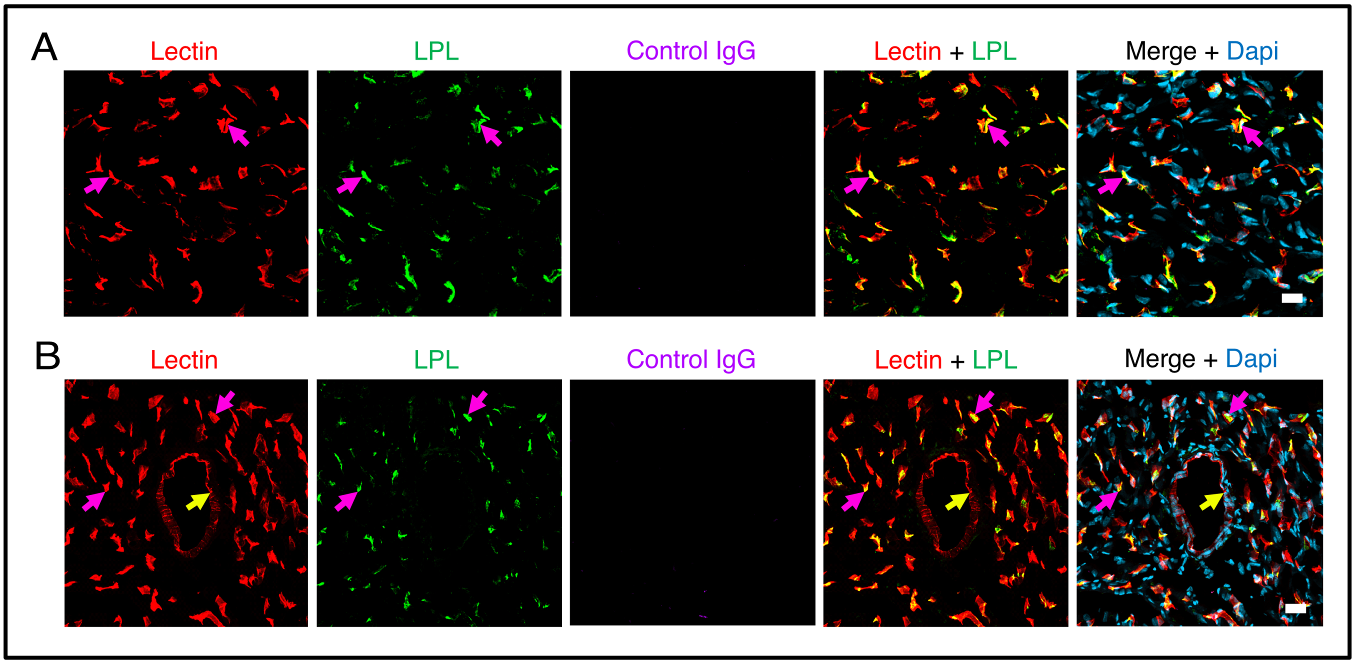

Supplemental Figure 4. Confocal micrographs showing that LPL in the chicken heart is located on the luminal surface of capillaries but not larger blood vessels. The chicken had been injected intravenously with a fluorescein-labeled lectin (*Lens culinaris agglutinin*, *red*), a rabbit antibody against chicken LPL (*green*), and an irrelevant hamster IgG (*purple*) (to demonstrate effective perfusion of the vasculature). The sections were then stained with an Alexa Fluor 647–conjugated secondary antibody against rabbit IgG and an Alexa Fluor 568–conjugated antibody against hamster IgG. Nuclei were stained with Dapi (*blue*). (**A**) Confocal micrograph showing that chicken LPL is on the luminal surface of lectin-stained capillary ECs *pink* arrows). Scale bar, 20 µm. (**B**) Confocal micrograph showing that chicken LPL is detectable in capillaries (*pink* arrows) but was not on a larger blood vessel (*yellow* arrow). Shown are representative images from experiments with 3 chickens. Scale bar, 20 µm.

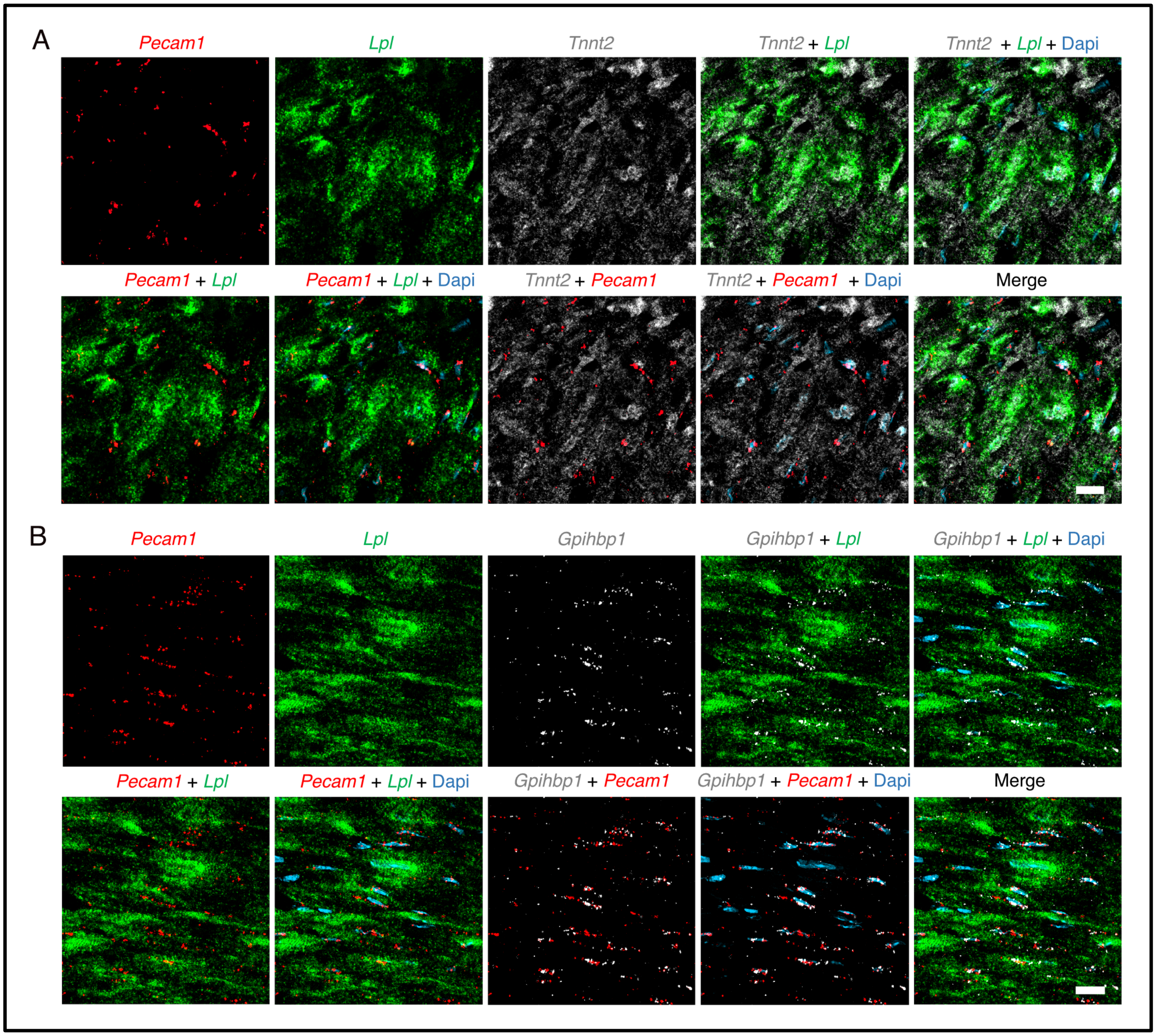

Supplemental Figure 5. ISH studies on mouse heart with *Lpl, Tnnt2, Pecam1, and Gpihbp1* RNAscope probes. (**A**) ISH studies of mouse heart revealing abundant amounts of *Lpl* transcripts *(green)* and *Tnnt2* transcripts (*white*) in cardiomyocytes; transcripts for *Pecam1* (*red*) were located in capillary ECs adjacent to cardiomyocytes. Scale bar, 20 µm. (**B**) ISH studies of mouse heart revealing abundant amounts of *Lpl* transcripts *(green)* in cardiomyocytes; *Pecam1* transcripts (*red*) and *Gpihbp1* transcripts (*white*) were in capillary ECs adjacent to cardiomyocytes. Nuclei were stained with Dapi (*blue*). Scale bar, 20 µm.

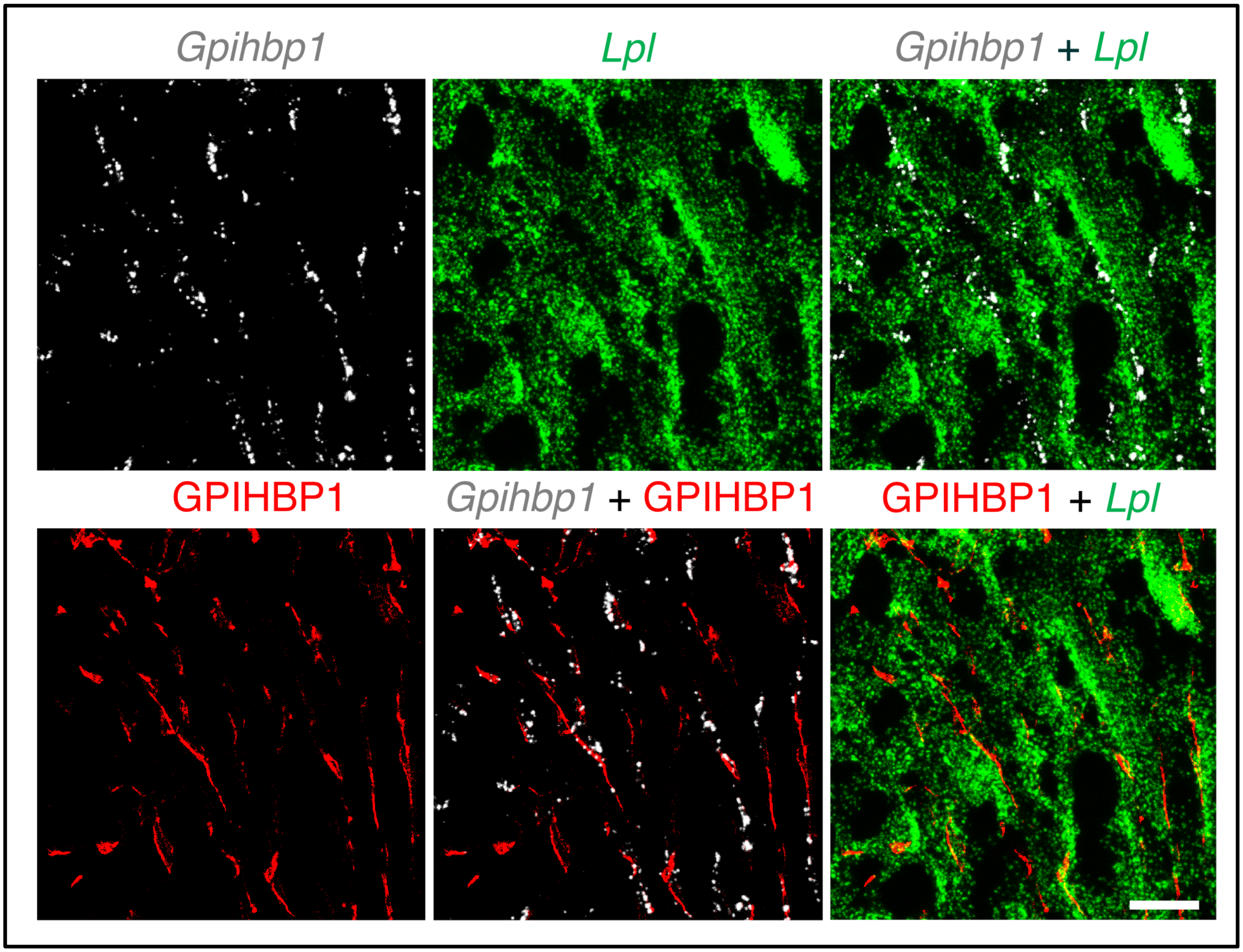

Supplemental Figure 6. A combined ISH/immunohistochemistry study of mouse heart showing that the distributions of *Gpihbp1* transcripts and GPIHBP1 protein are distinct from the distribution of *Lpl* transcripts. *Gpihbp1* transcripts (*white*) and *Lpl* transcripts (*green*) were detected with RNAscope probes; GPIHBP1 protein (*red*) was detected with a rat monoclonal antibody against mouse GPIHBP1 (11A12). *Lpl* transcripts were abundant in cardiomyocytes; *Gpihbp1* transcripts (*white*) and GPIHBP1 protein (*red*) were in capillaries adjacent to cardiomyocytes. Scale bar, 20 µm.

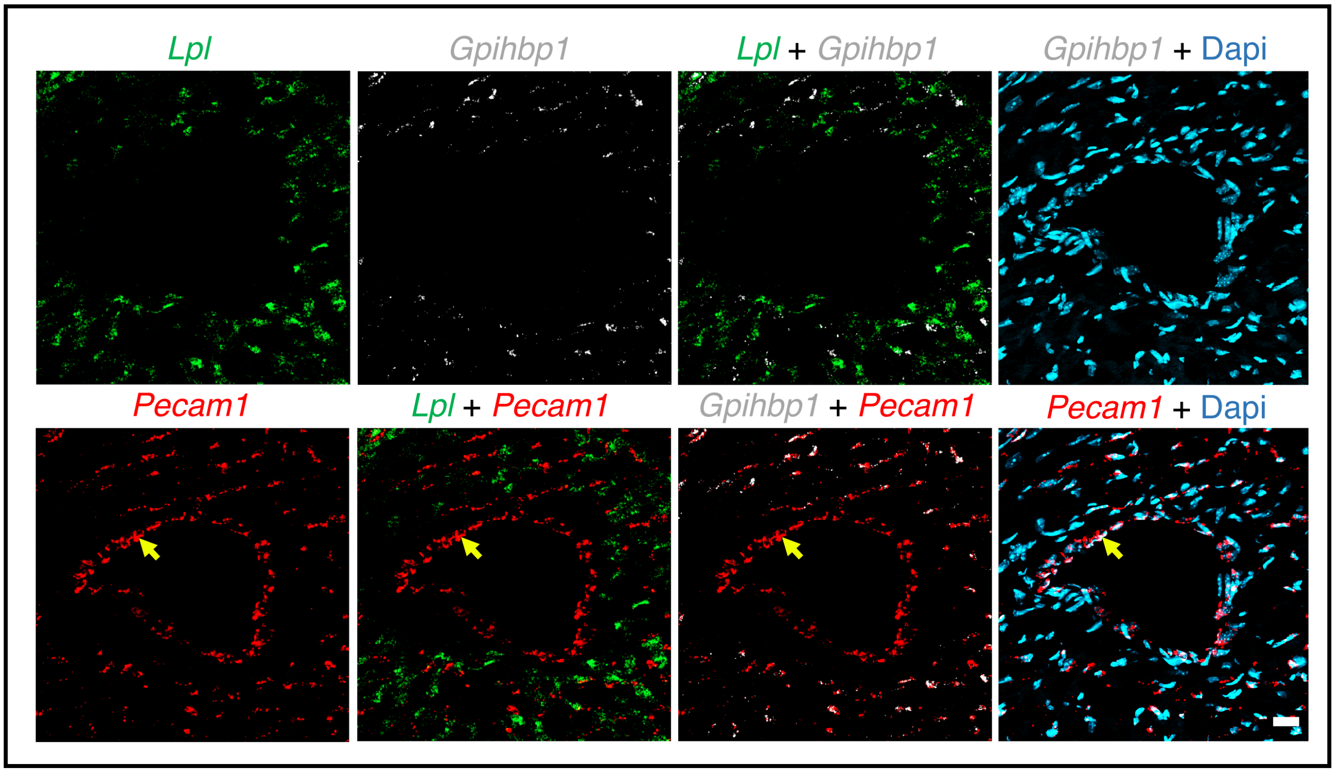

Supplemental Figure 7. ISH studies of mouse heart with RNAscope probes for *Lpl* (*green*), *Gpihbp1* (*white*), and *Pecam1* (*red*). *Gpihbp1* and *Pecam1* transcripts were in capillary ECs adjacent to cardiomyocytes (which contained large amounts of *Lpl* transcripts). *Pecam1* transcripts were also detected in ECs of a large blood vessel (*yellow* arrow). Nuclei were stained with Dapi (*blue*). Scale bar, 20 µm.

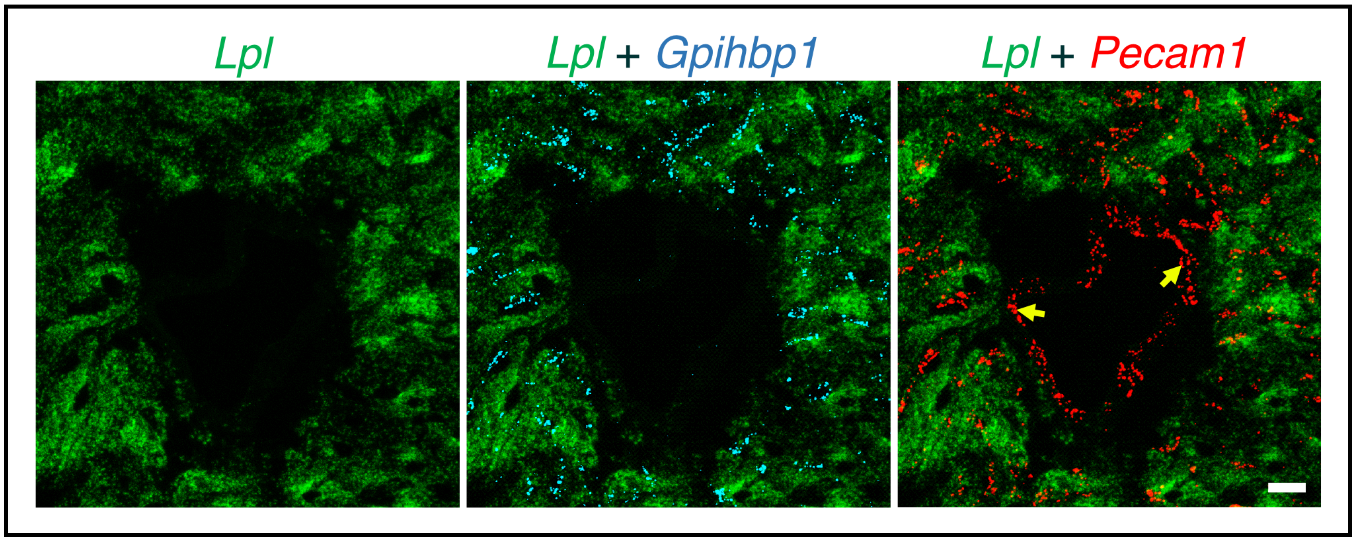

Supplemental Figure 8. ISH studies of mouse heart with RNAscope probes for *Lpl* (*green*), *Gpihbp1* (*blue*), and *Pecam1* (*red*). *Gpihbp1* and *Pecam1* transcripts were in capillary ECs adjacent to cardiomyocytes (which contained abundant *Lpl* transcripts). *Pecam1* transcripts were also detected in ECs of a large blood vessel (*yellow* arrows). Scale bar, 20 µm.

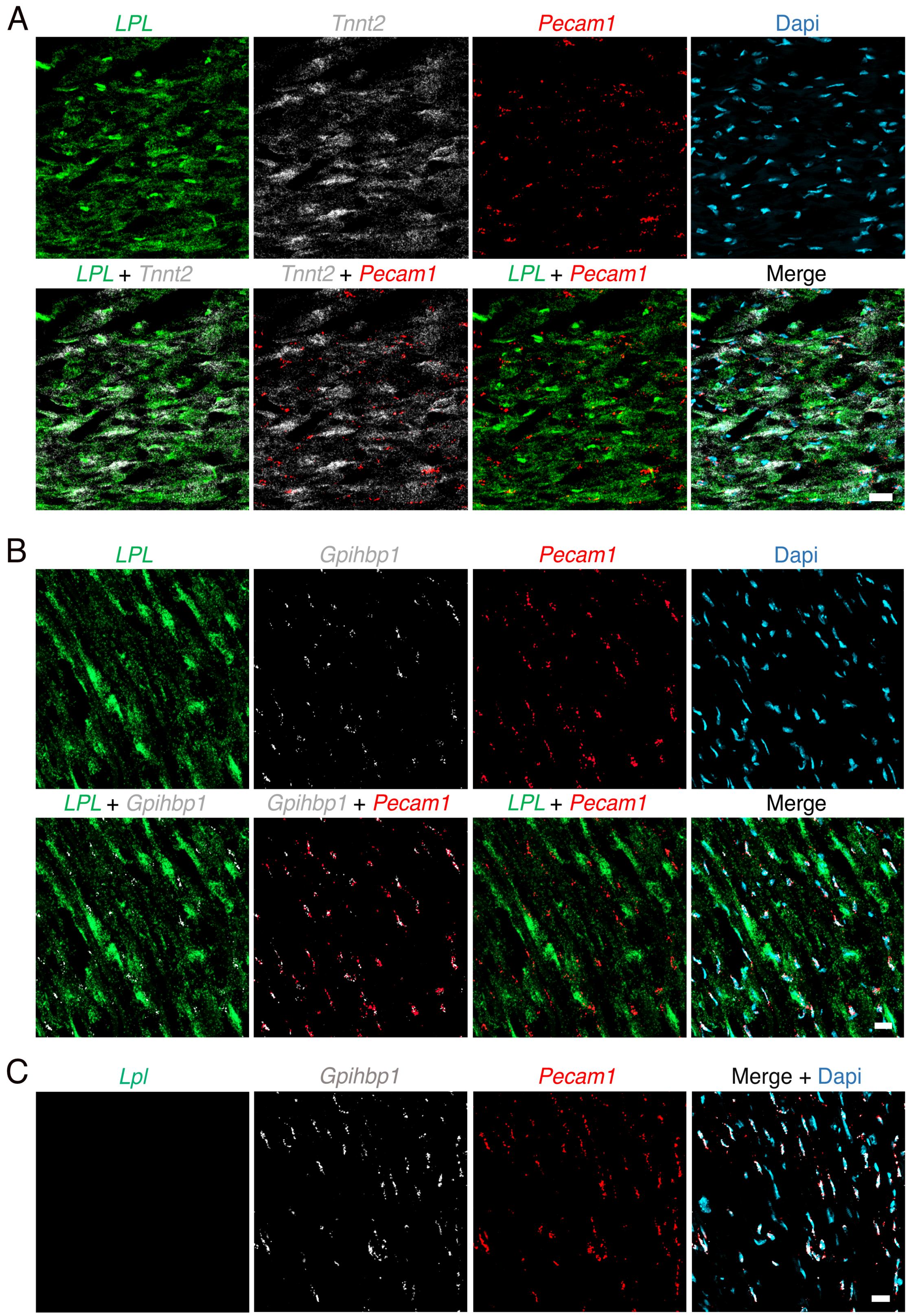

Supplemental Figure 9. ISH studies of heart from a *Lpl*$^{-/-}$MCK–hLPL mouse with RNAscope

probes. (**A**) ISH studies of cryosections of the heart from an *Lpl* $^{-/-}$MCK–hLPL mouse, revealing abundant amounts of human *LPL* transcripts *(green)* and *Tnnt2* transcripts (*white*) in cardiomyocytes; *Pecam1* transcripts (*red*) were in capillary ECs adjacent to the cardiomyocytes. Scale bar, 20 µm. (**B**) ISH studies of a *Lpl* $^{-/-}$MCK–hLPL mouse heart revealing abundant amounts of human *LPL* transcripts *(green)* in cardiomyocytes; *Pecam1* transcripts (*red*) and *Gpihbp1* transcripts (*white*) were in capillary ECs adjacent to cardiomyocytes. Nuclei were stained with Dapi (*blue*). Scale bar, 20 µm. (**C**) Mouse *Lpl* transcripts *(green)* were undetectable in hearts of *Lpl* $^{-/-}$MCK–hLPL mice; *Gpihbp1* (*white*); and *Pecam1* transcripts (*red*) were in capillary ECs. Scale bar, 20 µm.

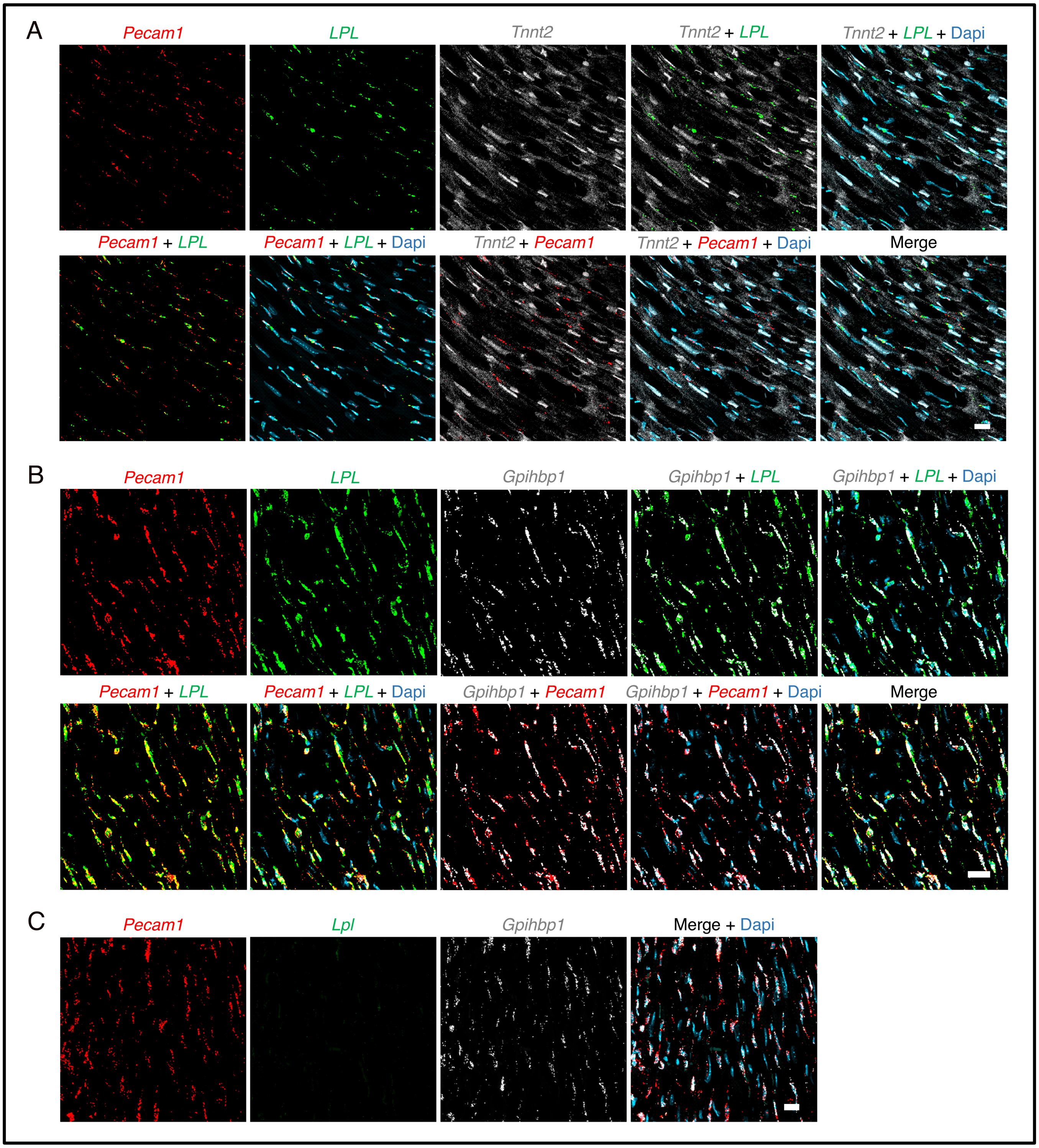

Supplemental Figure 10. Transcripts for human *LPL*, *Gpihbp1,* and *Pecam1* are in heart capillary ECs of *Lpl* $^{-/-}$Tie2–hLPL mice. (**A**) ISH studies of the heart from an *Lpl* $^{-/-}$Tie2–hLPL mouse, revealing abundant *Tnnt2* transcripts (*white*) in cardiomyocytes; *Pecam1* transcripts (*red*) and *LPL* transcripts (*green*) were in capillary ECs adjacent to cardiomyocytes. Scale bar, 20 µm. (**B**) ISH studies of heart from an *Lpl* $^{-/-}$Tie2–hLPL mouse, revealing *Pecam1* transcripts (*red*), human *LPL* transcripts (*green*), and *Gpihbp1* transcripts (*white*) in capillary ECs. Nuclei were stained with Dapi (*blue*). Scale bar, 50 µm. (**C**)

Mouse *Lpl* transcripts *(green)* were undetectable in hearts of *Lpl* $^{-/-}$Tie2–hLPL mice; *Gpihbp1* (*white*) and *Pecam1* transcripts (*red*) were in capillary ECs. Nuclei were stained with Dapi (*blue*). Scale bar, 20 µm.

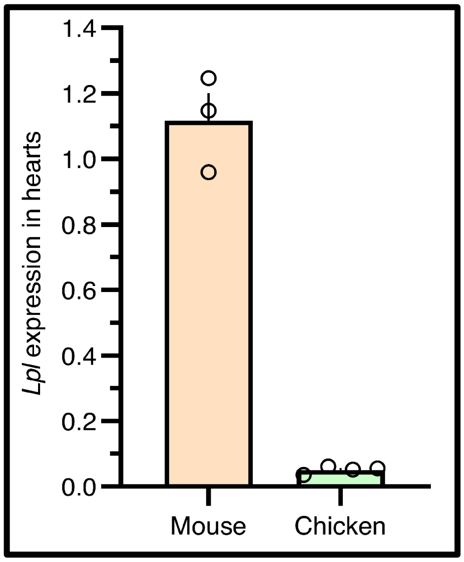

Supplemental Figure 11. *Lpl* transcript levels, relative to *Gapdh* transcript levels, in mouse (*n* = 3) and chicken (*n* = 4) hearts. Chicken and mouse *Lpl transcript levels* (normalized to *Gapdh*) were measured by qRT-PCR studies with primers corresponding to perfectly conserved sequences in mouse and chicken *Lpl* and *Gapdh* transcripts. Mean ± SEM; ***P < 0.001.

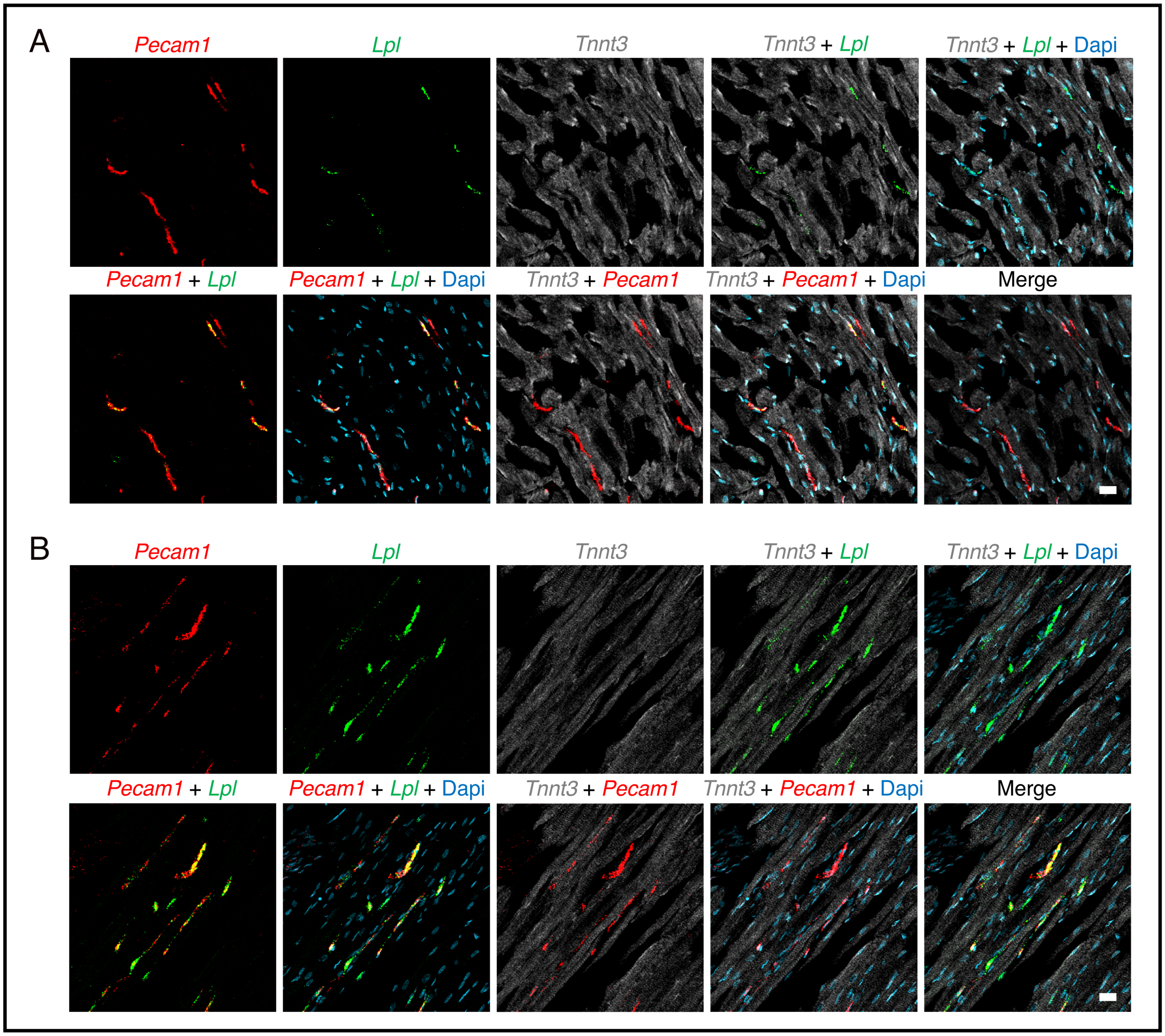

Supplemental Figure 12. ISH studies of *pectoralis major* (A) and *quadriceps* (B) of a 5-day-old chicken with RNAscope probes for *Lpl* (*green*), *Pecam1* (*red*) and *Tnnt3* (*white*). *Lpl* and *Pecam1* transcripts were in capillary ECs adjacent to myocytes (*Tnnt3*, *white*). Nuclei were stained with Dapi (*blue*). Scale bars, 20 μm.

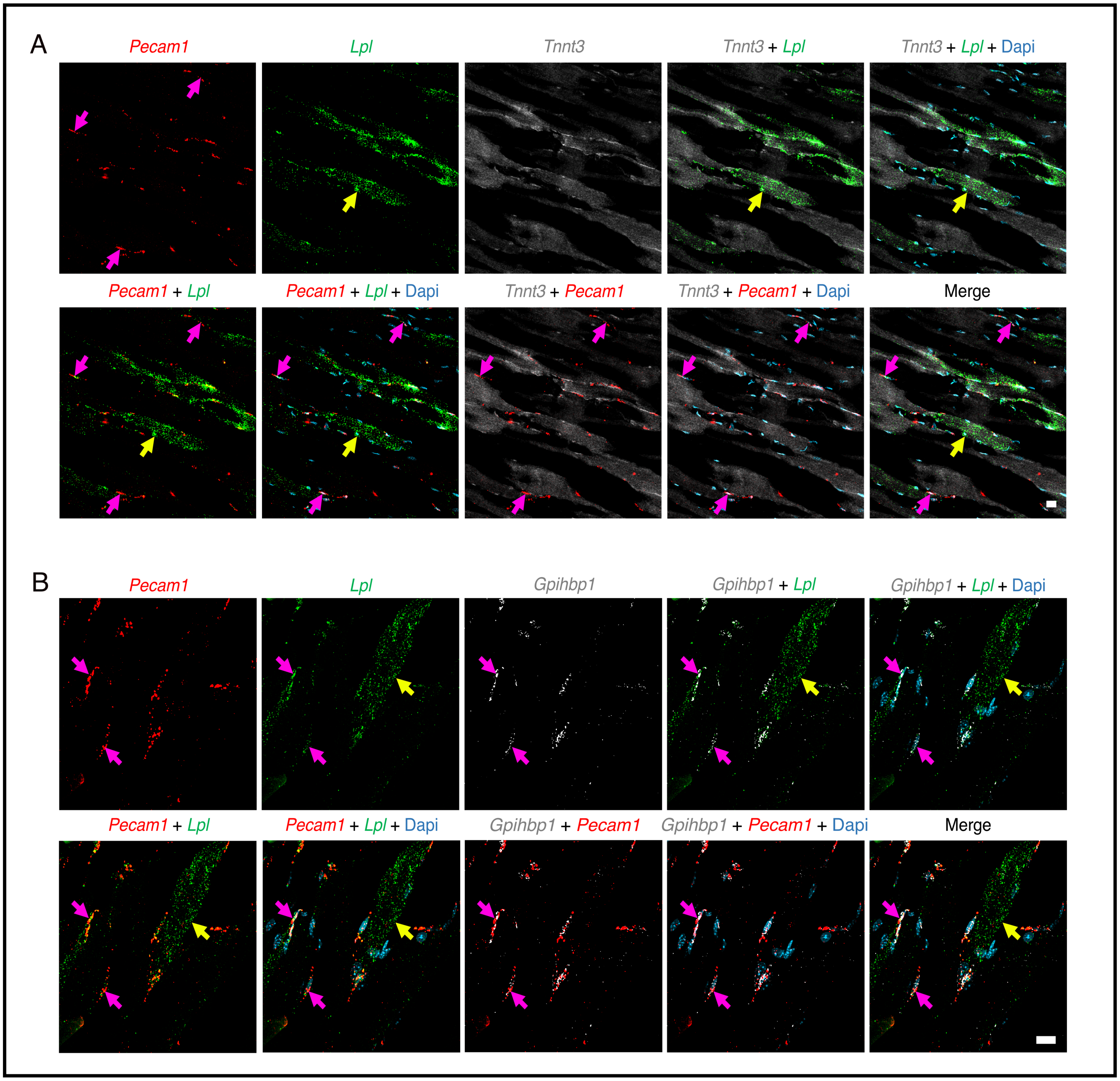

Supplemental Figure 13. ISH studies of mouse skeletal muscle RNAscope probes. (**A**) ISH study of *quadriceps*, which contains a mixture of red and white muscle fibers, with for probes for *Pecam1* (*red*), *Lpl* (*green*), and *Tnnt3* (*white*). *Lpl* transcripts were abundant in a subset of myocytes (*yellow* arrows); myocytes were identified by *Tnnt3* transcripts. Muscles containing red muscle fibers express high levels of LPL, whereas muscles containing white muscle fibers express low levels of LPL (MH Tan et al. *J Lipid Res.* 1977;18(3):363-7). *Lpl* transcripts were detectable at lower levels in *Pecam1*-positive capillary ECs (*pink* arrows). (**B**) ISH study of *quadriceps* with probes for *Pecam1* (*red*), *Lpl* (*green*), and *Gpihbp1* (*white*). *Lpl* transcripts were abundant in a subset of myocytes (*yellow* arrows). Low levels of *Lpl* transcripts were detectable in *Pecam1*- and *Gpihbp1*-positive capillary ECs (*pink* arrows). Nuclei were stained with Dapi (*blue*). Scale bars, 20 µm.

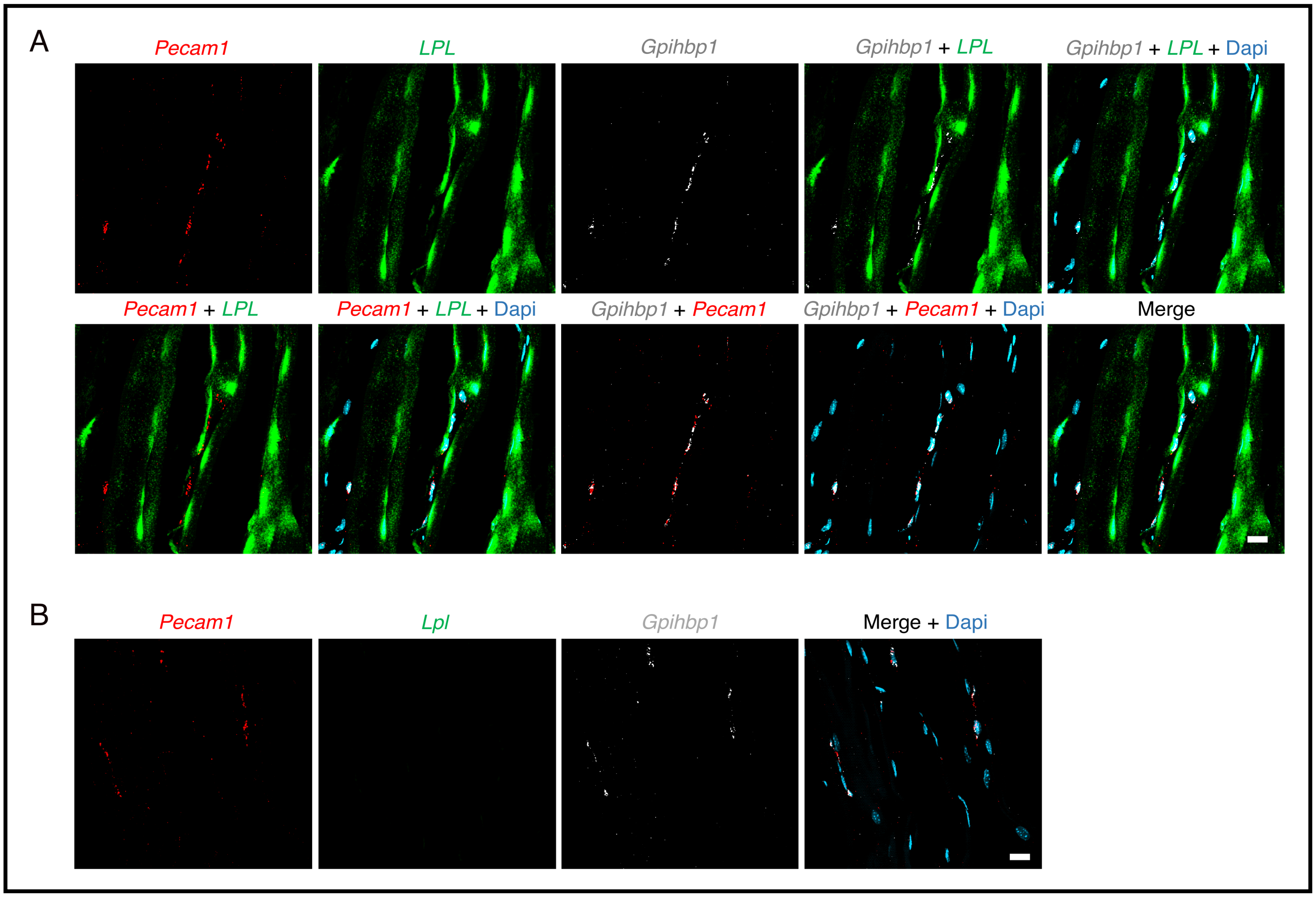

Supplemental Figure 14. ISH studies of *quadriceps* from an *Lpl*$^{-/-}$MCK–hLPL mouse with RNAscope probes. (**A**) ISH studies with RNAscope probes for *Pecam1* (*red*), human *LPL* (*green*), and *Gpihbp1* (*white*). Human *LPL* transcripts were abundant in skeletal myocytes; *Pecam1* and *Gpihbp1* transcripts were in capillary ECs. Scale bar, 20 µm. (**B**) ISH studies with RNAscope probes for *Pecam1* (*red*), mouse *Lpl* (*green*), and *Gpihbp1* (*white*). As expected, mouse *Lpl* transcripts were undetectable; *Gpihbp1* and *Pecam1* transcripts were in capillary ECs. Scale bar, 20 µm.

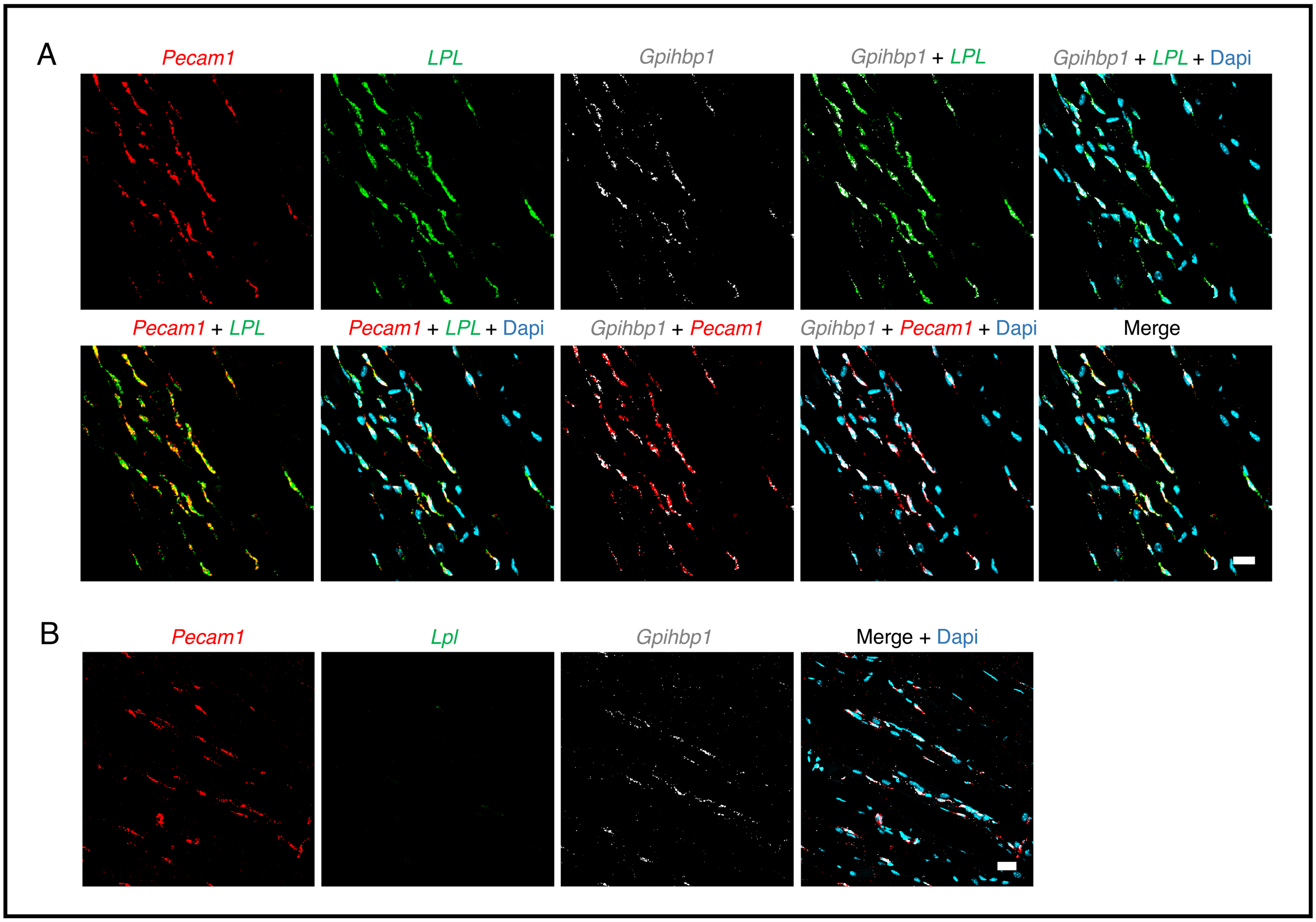

Supplemental Figure 15. ISH studies of *quadriceps* from an *Lpl*$^{-/-}$Tie2–hLPL mouse with RNAscope probes. (**A**) ISH studies revealed *Pecam1* (*red*), human *LPL* (*green*), and *Gpihbp1* (*white*) expression in capillary ECs. Nuclei were stained with Dapi (*blue*). Scale bar, 20 µm. (**B**) ISH studies revealed that mouse *Lpl* transcripts *(green)* were undetectable in the *quadriceps* of *Lpl*$^{-/-}$Tie2–hLPL mice; *Gpihbp1* (*white*) and *Pecam1* transcripts (*red*) were located in capillary ECs. Nuclei were stained with Dapi (*blue*). Scale bar, 20 µm.

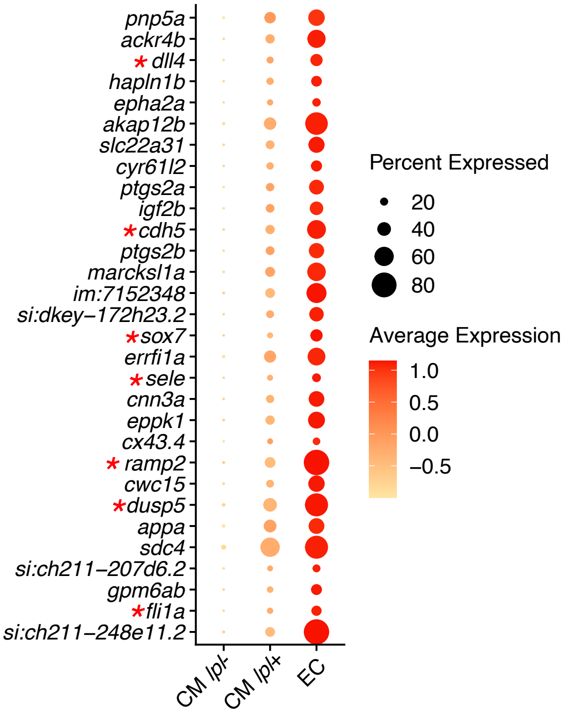

Supplemental Figure 16. Analysis of single-cell RNA transcriptomic studies on zebrafish heart, revealing substantial endothelial cell gene expression in a subset of cardiomyocytes (2.4% of the total) that contain *lpl* transcripts. Shown here are the top 30 genes enriched in *lpl*+ cardiomyocytes compared with *lpl*– cardiomyocytes (Wilcoxon rank-sum tests). All of the genes listed here are expressed highly in ECs compared with cardiomyocytes, and several are well-established EC markers (*). For each of the 30 genes, the expression levels in *lpl*+ vs. *lpl*– cardiomyocytes were markedly different ($P < 0.00001$ after correction for multiple tests). These studies show that the presence of *lpl*+ cardiomyocytes in the zebrafish reflects EC contamination.

Table S1. PCR oligonucleotide primer sequences corresponding to perfectly conserved sequences in mouse and chicken *Lpl* and *Gapdh*.

Targets	Forward (5′→3′)	Reverse (5′→3′)
Mouse and chicken *Lpl*	CAGCTGGTGAAGTGCTC	ACTTTGTAGGGCATCTGAG
Mouse and chicken *Gapdh*	CCATGTTTGTGATGGGTGT	AATGCCAAAGTTGTCATGGA
