# Supplementary material for: Distinct strategies for intravascular triglyceride metabolism in hearts of mammals and lower vertebrate species
Source: JCI Insight. 2024 Sep 17;9(20):e184940. doi: 10.1172/jci.insight.184940 (PMC11529983; doi:10.1172/jci.insight.184940)

Full unedited blot for:

Figure S1 in the *JCI* submission, "Strikingly different patterns of LPL expression in hearts of mammals and lower vertebrates," by Nguyen *et al.*

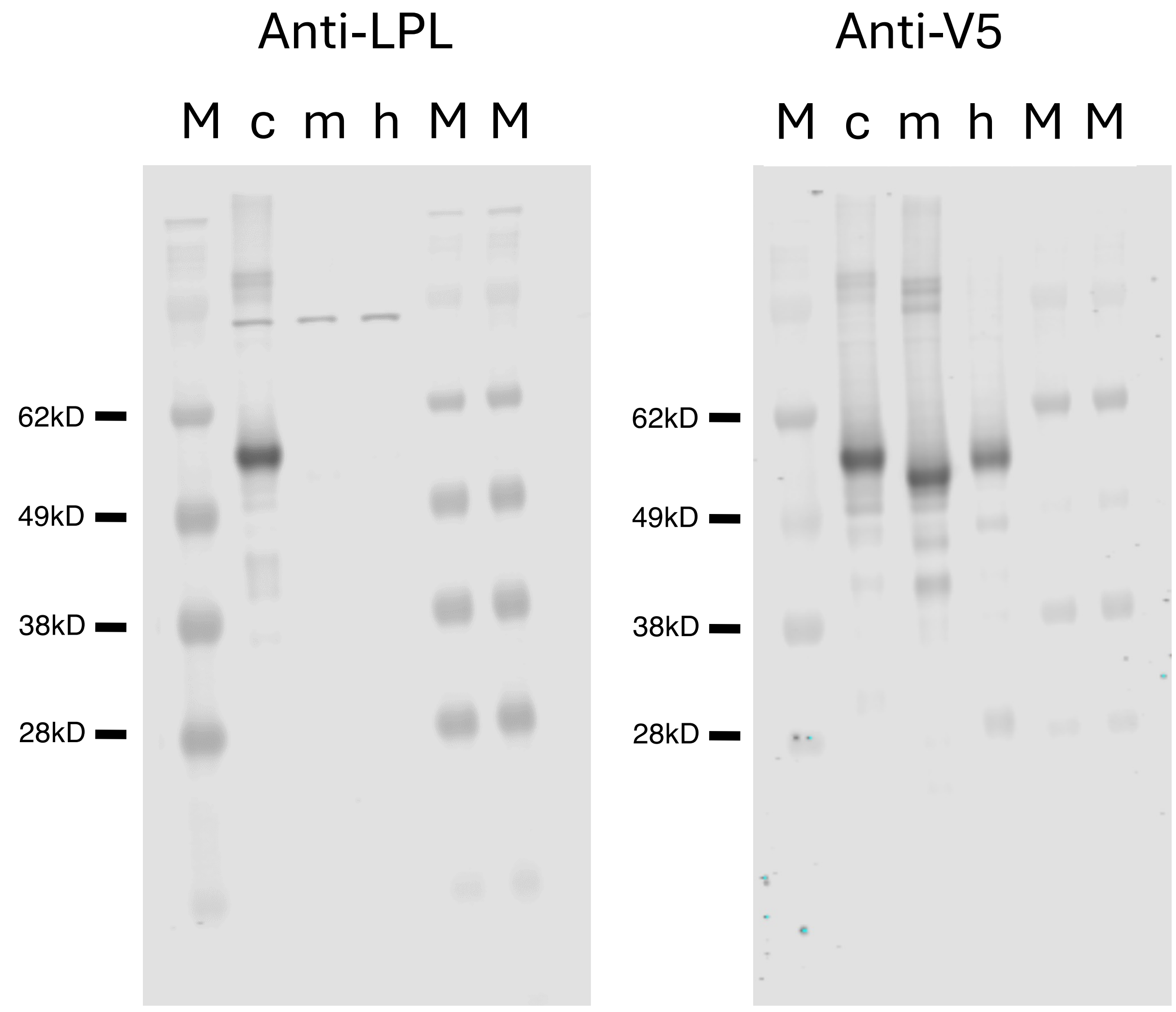

Supplement: Unedited blot and gel images [file jciinsight-9-184940-s173.pdf]
